# RankProt: A multi criteria-ranking platform to attain protein thermostabilizing mutations and its *in vitro* applications - Attribute based prediction method on the principles of Analytical Hierarchical Process

**DOI:** 10.1371/journal.pone.0203036

**Published:** 2018-10-04

**Authors:** Debamitra Chakravorty, Sanjukta Patra

**Affiliations:** Department of Biosciences and Bioengineering, Indian Institute of Technology Guwahati, Guwahati, Assam, India; Universidade Nova de Lisboa Instituto de Tecnologia Quimica e Biologica, PORTUGAL

## Abstract

Attaining recombinant thermostable proteins is still a challenge for protein engineering. The complexity is the length of time and enormous efforts required to achieve the desired results. Present work proposes a novel and economic strategy of attaining protein thermostability by predicting site-specific mutations at the shortest possible time. The success of the approach can be attributed to Analytical Hierarchical Process and the outcome was a rationalized thermostable mutation(s) prediction tool- RankProt. Briefly the method involved ranking of 17 biophysical protein features as class predictors, derived from 127 pairs of thermostable and mesostable proteins. Among the 17 predictors, ionic interactions and main-chain to main-chain hydrogen bonds were the highest ranked features with eigen value of 0.091. The success of the tool was judged by multi-fold *in silico* validation tests and it achieved the prediction accuracy of 91% with AUC 0.927. Further, *in vitro* validation was carried out by predicting thermostabilizing mutations for mesostable *Bacillus subtilis* lipase and performing the predicted mutations by multi-site directed mutagenesis. The rationalized method was successful to render the lipase thermostable with optimum temperature stability and *T_m_* increase by 20°C and 7°C respectively. Conclusively it can be said that it was the minimum number of mutations in comparison to the number of mutations incorporated to render *Bacillus subtilis* lipase thermostable, by directed evolution techniques. The present work shows that protein stabilizing mutations can be rationally designed by balancing the biophysical pleiotropy of proteins, in accordance to the selection pressure.

## Introduction

Living things evolve naturally with mutation as the tool to survive in various selection pressures-such as extreme temperature. Though thermostable proteins have various industrial applications, culturing them in laboratory from their natural sources is a daunting task [1]. Thus, spectrum of research efforts is on continuous expansion to attain protein thermostability by *in vitro* mutations. To achieve successful thermostabilizing mutations many researchers tried to understand the underlying mechanisms behind protein thermostability [2–5]. The conclusion was that a particular rule does not exist for predicting thermostabilising mutations and thermostability is said to be a compound effect of various protein stabilising factors [2, 6]. Conventionally, researchers have applied basically three approaches to attain higher protein thermostability. First, directed evolution strategy was developed-mimicking nature. The process involves introduction of random mutations in recombinant protein clones, followed by colony screening of the desired traits [7–8]. By this method, till date, increase in thermostability of up to 35°C have been attained [9]. However, the main drawback is that the effect of mutations on the stability of protein is challenging to predict *a priori*. Further the protocol followed is generally laborious and protracted, often leading to screening of up to 10^8^ colonies of expression hosts to identify stable constructs [9]. A good comparison of the success rate of such methods has been reported recently by Bednar et al. (2015). They showed that methods involving colony screening achieve only a 0.1% success rate and experimental statistics reveal that only 0.5–0.01% of random mutations achieved by such methods are actually beneficial [9]. The second approach was the ancestral or consensus methods [10–12]. This method involves substitution in the subject protein with conserved amino acid residues present in the fitter ancestral protein. The success of the method relies on multiple sequence alignments and availability of a highly homologous ancestral sequence hinders the broad applicability of the method. The third and the most promising approach is protein structure guided methods. Recent work by Sikosek et al. in 2014 reported that there are experimental evidences which positively correlate between protein function and its stability [13]. The fate of mutations can be small or large depending on the position where the mutation has occurred and its ability to modulate various intra- and inter-protein attributes resulting in a better stabilised protein [14]. For example, Eijsink et al. 1992 improved thermostability of neutral proteinase of *Bacillus stearothermophilus* by enhancement of hydrogen bonding networks [15]. In another work, surface electrostatic interactions were improved in the cold shock, RNase T1, and CheY proteins to enhance their thermostability after screening 251 and 244 single-mutant structures respectively [16]. Irrespective of the success of this approach, it suffers when few biophysical properties like disulphide bonds or ionic interactions are targeted for modulation by mutations, without prior understanding of their effect on the mutated protein [17, 18]. Again the approach can fail if it results in incorporation of mutations which are self-compensatory leading to no additive effect on protein stability [19].

Therefore, experimentally, these three approaches have their own practical limitation in their global applicability to enhance thermostability of any preferred protein. Hence, a faster and more powerful approach to render proteins thermostable was desirable. This necessitated prediction of point mutations that enhance protein thermostability, prior to performing site directed mutations and identification of stable constructs. In this direction, up to now, many computational algorithms are available that predict point mutations which can enhance the overall thermodynamic stability of proteins (for details about their performance, refer to [20–29]. Though the algorithms have broad applicability by predicting stabilizing mutations, most of them predict thermodynamic stability. Whereas, thermal stability (dependent on melting temperature, *T_m_*) changes upon point mutations, have been less investigated [30]. Most of such algorithms were also reported to be moderately accurate in predicting protein stability by Khan et al., (2010), who compared 11 such online stability predictors [31]. Further it was reported that when the predicted single point stabilising mutations were recombined to provide additive effects, it often resulted in antagonistic epistatic effects of individual mutations [32]. It was also reported that the mutants obtained by computational methods rarely showed increase greater than 15°C of melting temperature (*T_m_*) [7, 9]. Again the success rate of these methods were predicted to be <50% for prediction of stabilizing single point mutations and >50% success for methods that incorporate the effect of multiple mutations [9]. In this direction, only two algorithms have been developed that can predict stabilising multi- point mutations, namely ERIS and WET [33–34]. However, the accuracy of WET was reported to drop to 0.57 when it was tested on the hypothetical reverse mutations [4]. Thus, promiscuity in these reported approaches raises difficulty to scoop out the thermostabilizing mutations in laboratory.

In this work to render the process of attaining thermostable proteins more comprehensive, predicting multiple thermostabilizing mutations is what the authors intended. We propose a novel method to predict thermostabilizing mutations, by ranking intra-protein tertiary interactions, employing Analytical Hierarchical Process. This was carried out by collection of robust binary protein dataset of thermostable and mesostable homologous protein pairs, prioritizing multiple protein biophysical features and development of a thermostabilizing mutation prediction tool. The tool was validated in a multifold manner to prove rationality and accuracy. The developed route was successful to circumvent the requirement of selection pressure and colony screening, ensuring results in the shortest possible time by utilization of lesser effort and capital in comparison to the existing methods.

## Methods

### Dataset creation and feature selection

**Fig 1:**
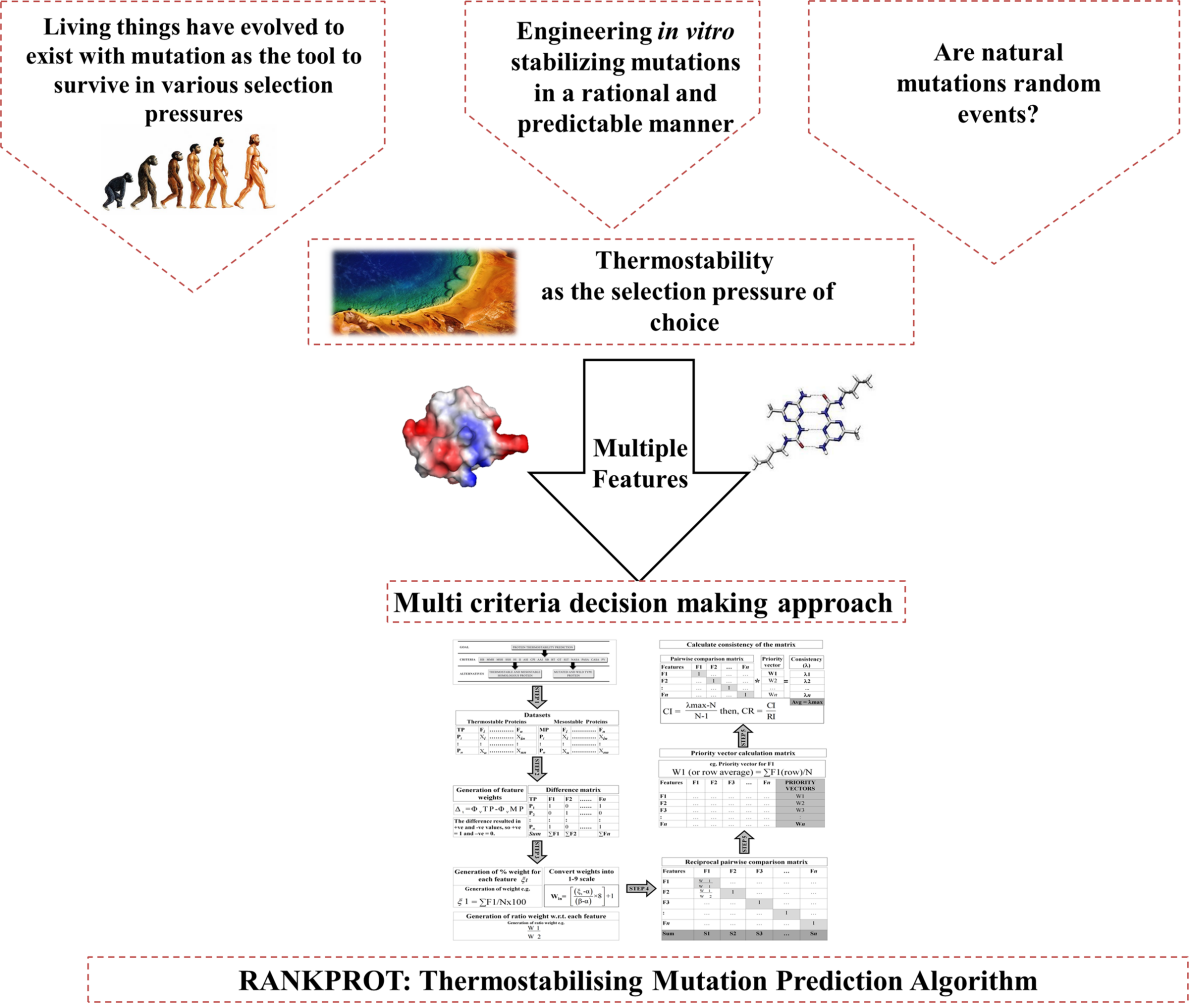
Overview of the method of developing RankProt with thermostabiility as the selection pressure of choice. The line drawings were made by the authors. All other images in this figure were adapted from Wikipedia possessing CC BY-SA 3.0 license.

With thermostability as the selection pressure, the data collection pertained to thermostable protein structures. The overview of the method is illustrated in Fig 1. Initial key words search of “thermostable”, “thermophilic” and “hyperthermophilic” resulted in 1280 structures from RCSB PDB. After removing partially sequenced proteins and putative sequences, 378 thermostable protein structures were retained. Thermostability is relative to mesostability hence, the requirement of mesostable homologus counterparts. Mesostable counterparts were chosen by BLAST search. Only structural protein pairs with sequence similarity >70% and RMSD <2Å were retained. The final data set contained 127 non-redundant homologous thermophilic-mesophilic protein pairs (S1 Table). The spectrum of data pertained to the entire phylogeny and all six enzyme classes with inclusion of the structural proteins. This reflects the global phylogenetic distribution and unbiased nature of the dataset. An in house tool developed on python platform enumerated all the biophysical features pertaining to intra-protein non-covalent interactions. The criteria for the cut-off lengths to be treated as a yardstick were taken from literature. The details of these have been presented in S2 Table. PROMOTIF and Volume Area Dihedral Angle Reporter (VADAR) were integrated with the aforementioned code [35–36]. This rendered us with twenty-five protein structural features. All features were the count of the number of interactions in proteins. The calculation pertaining to surface areas were represented as fractions out of the total accessible surface area of proteins. All calculated features were normalized with respect to the number of atoms or the length of the protein sequence and further converted to their percentage scores. Features were initially filtered through non parametric two-tail Kolmogorov Smirnov (KS) test. Significant features having a p value <0.05 were chosen. In a bipartite dataset, the significance was based on the calculation of D-statistic that states significant difference between the distribution for the thermostable and mesostable groups of protein. Two tail KS test have been used previously by many researchers for feature selection [37–44]. This resulted in final set of 17 features (Table 1). After feature generation and partial selection by two tail KS test, it was necessary to know the significance of each feature contributing to thermostability. The problem in hand involved multiple features. Hence, a robust method that can handle all such features simultaneously was required. We proceeded with Multi Criteria Decision Making approach.

**Table 1:** The statistically significant features contributing to thermostability obtained by two-tail Kolmogorov Smirmov-test p<0.05.

Sl. No	Number of Interactions	Abbreviation	Software/ Tools
1	Percentage of residues forming Hydrogen bonds	HB	VADAR
2	Main chain to main chain hydrogen bonds	MMH	IPI enumerator*
3	Main chain to side chain hydrogen bonds	MSH	IPI enumerator
4	Side chain to side chain hydrogen bonds	SSH	IPI enumerator
5	Hydrophobic interactions	HI	IPI enumerator
6	Ionic interactions	II	IPI enumerator
7	Aromatic-sulphur interaction	ASI	IPI enumerator
8	Cation-pi interactions	CPI	IPI enumerator
9	Aromatic-aromatic interactions	AAI	IPI enumerator
10	Salt bridge	SB	IPI enumerator
11	Packing volume	PV	VADAR
12	Beta turns	BT	PROMOTIF
13	Total gamma turns	GT	PROMOTIF
14	Inverse gamma turns	IGT	PROMOTIF
15	Fraction non polar Accessible surface area (ASA)	NASA	VADAR
16	Fraction polar ASA	PASA	VADAR
17	Fraction charged ASA	CASA	VADAR

*In house developed tool in python. The cut-offs taken for the calculations are the standards available in literature. The details of these have been presented in S2 Table.

### Ranking features contributing towards protein thermostability

**Fig 2:**
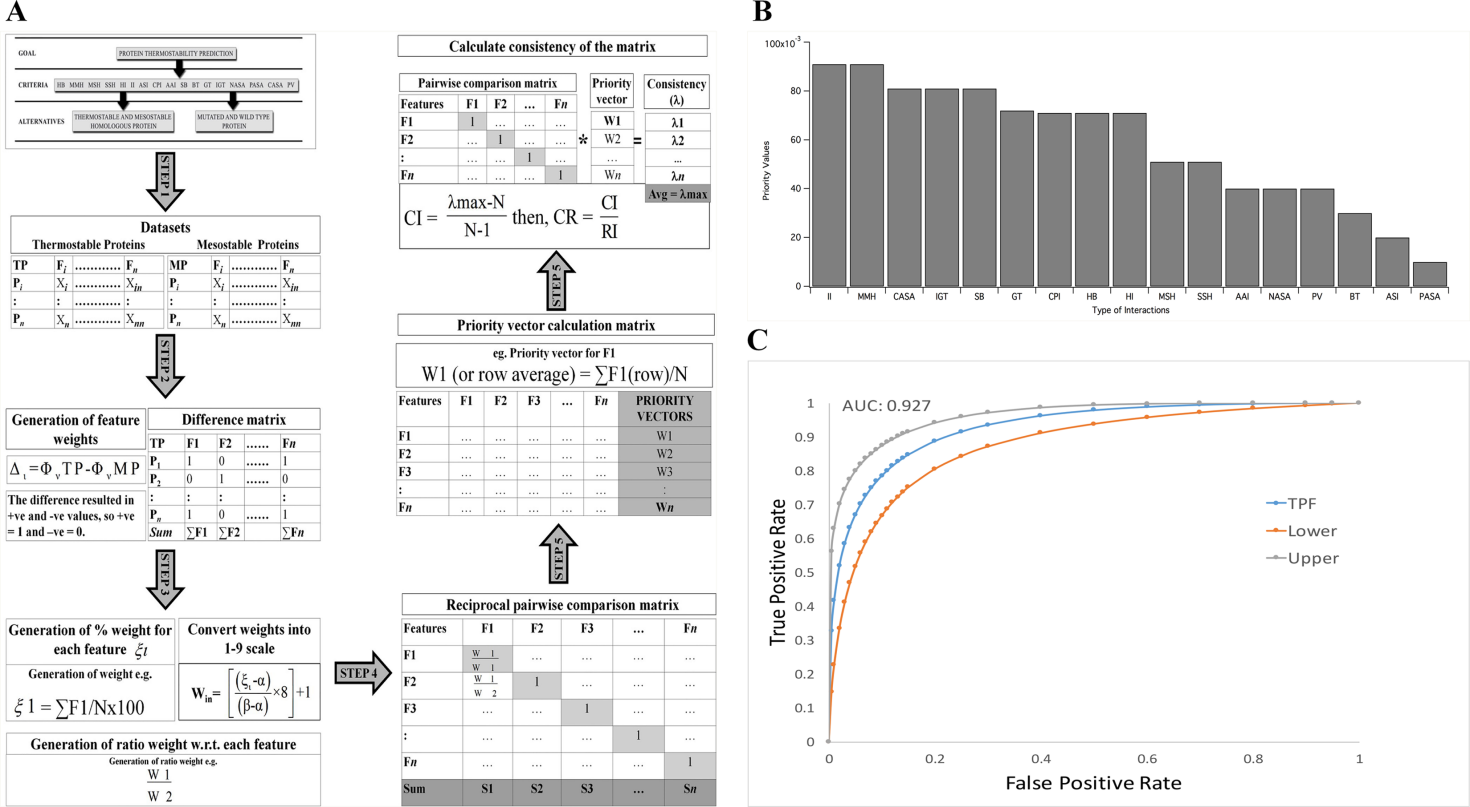
The rationalized method for predictioin of thermostability of proteins. A The hierarchy of thermostability is composed of three tiers. First tier is the goal and second is the criteria. The third tier was the alternatives. The alternatives are a set of wild type or engineered proteins. In Step 1 the 127 thermostable and mesostable proteins along with their 17 features form a 127x17 matrix, where TP: Thermostable proteins; MP: Mesostable proteins; F: features where i⋯n represents number of features; P: Protein ID where i⋯n stands for the number of proteins. In Step 2 feature weights are generated where, Δ_l: difference in the normalized feature; l = i⋯n features, ΦTP: normalized feature of the TP set; Φ_(v) MP: normalized feature of the MP set. Then the difference in the features was represented by vectors where each difference in attribute, Δ_l takes the value of 1 if the feature of type F is positive, and 0 if F is negative or there is no difference. Further the number of proteins in the difference matrix having the value of 1 for each of the features was summed. In Step 3, percentage weight showing increase, ξι w.r.t. the total number of thermostable proteins, N = 127, and ι = 1⋯n features was derived and converted into the 1–9 scale where, W_in is the converted weight, in = 1⋯n features, α is the minimum value in the weight for feature and β is the maximum value in the weight of feature. This weight was further converted to ratio weight for each feature. In Step 4 this ratio was supplied to a pairwise comparison matrix. In Step 5, calculation of eigen or priority vectors was performed by calculating the sum of the column Sn where n = 1⋯n features. In Step 6, Consistency index (CI) was derived by calculating λmax which is the consistency measure of each row which is calculated as the dot product of pairwise comparison matrix with the priority vector matrix which is then divided by N, where N = total number of features. Further, consistency ratio (CR) was derived and according to Alonso et al. (2006), RI is a random index and is equal to 1.6086 for N = 17. B The priority values obtained by AHP for the 17 significant thermostabilizing features. The scale is from 0–1. The highest ranked features were ionic interactions (II) and Main-chain–main-chain hydrogen bonds. The abbreviations have been provided in the beginning of the paper. (C) Receiver operating characteristic curve obtained by Leave one out Cross Validation, fitted by maximum likelihood estimation by LABROC4 for ranks of 100 thermostable proteins w.r.t. mesostable homologous proteins obtained by RankProt. The Area Under Curve (AUC) was calculated to be 0.927.

The heuristic model of Analytical Hierarchical Process (AHP) was employed to rank thermostabilizing features. The method has been used in industrial engineering applications, selecting ecological indicators for river flow restoration, and in health care research [43, 44]. Fig 2A illustrates the method developed to classify thermostable proteins and to identify thermostabilizing mutations. It involved four phases, the hierarchical structuring of complexity into homogeneous clusters of factors with objective i.e., classifying thermostable protein and prediction of thermostable mutations; criteria (17 statistically significant features) and alternatives (thermostable and mesostable proteins or mutated and wild type proteins). Secondly deriving at weights for criteria and alternatives and representing those with numbers. Thirdly, using the numbers to calculate the priorities of the criteria and alternatives and lastly completing the synthesis of these results to determine the most important alternative [45]. The derived features were numeric thus, pro-rata weights for the thermostability datasets were generated. Thus to prioritize the criteria we derived at their weights and formed a positive reciprocal pairwise comparison matrix which has been presented as supplementary material (S3 Table). Further steps as illustrated in Fig 2A led to the calculation of the priority vectors for the 17 features. The consistency of the method was calculated by deriving at consistency ratio and the matrix was accepted as consistent if CR < 0.158.

### Development of thermostabilising mutation(s) prediction tool: Rankprot

After feature ranking, the thermostability predictor-RankProt was written in Python. The principle was predicting thermostabilising mutations of a test protein by relatively ranking the mutated test protein (mutations carried out by *in silico* strategies) with respect to the non-mutated native protein, whose temperature stability is already known. This was achieved by calculating the dot product of 1x17 feature matrix generated for the test proteins by the 17x1 matrix of priority values of the 17 biophysical features obtained by AHP. The dot products were the relative normalized rank of the test and native proteins, summing to 1. This tool requires third party software VADAR and Promotif which are freely available. Details of the method are available with the tool; the source code can be downloaded from https://github.com/Debamitraiit/RankProt.

### Validating RankProt

Multi-fold validation of RankProt was carried out to check the applicability of the developed method. The first blind test was carried out by randomly choosing 100 thermostable-mesostable protein pairs from RCSB Protein Data Bank. These were processed using RankProt to derive at the ranks. A second blind test was carried out with a set of 40 mesostable proteins with another mesostable counterpart chosen randomly from the final dataset. The proteins were assigned ranks, the mean rank value and the pairwise difference were calculated for the thermostable-mesostable as well as mesostable-mesostable dataset. In the latter case, the pairs will not be homologous but both the proteins being mesostable was predicted to have marginal rank difference. For the former test set, the rank difference was predicted to be considerable. The next phase consisted of three more validity tests of RankProt. (I) All the mutated thermostable structures of *Bacillus subtilis* lipases were retrieved from RCSB Protein Data Bank and were ranked with respect to their wild type mesostable structure (PDB ID: 1i6w). (II) Mutated structures that led to gain of stability of bacteriophage T4 lysozyme (PDB ID: 2lzm) were ranked. Finally, (III) mutated structures of human lysozyme (PDB ID: 1lz1) that led to loss of stability as compared to wild type were ranked. The final performance of RankProt was analysed by calculating its accuracy. Where, accuracy is defined as number of correct assessments upon the number of all assessments. Receiver operating characteristic (ROC) curve was drawn and Area Under Curve (AUC) derived by fitted Maximum likelihood estimation by LABROC4 (http://www.jrocfit.org).

### Case study: Predicting thermostabilising mutations for *Bacillus subtilis* lipase

The final step in validating RankProt was by carrying out a case study. *Bacillus subtilis* lipase was the candidate of choice as the candidate lipase has all required features for qualifying as an industrially important catalyst. The sequence for the wild type lipase of *Bacillus subtilis* 168 (1i6w) was collected from Uniprot Knowledgebase release 2010_06 (Uniprot ID: P37957). Mutations were predicted using RankProt. To validate the stability of mutations, they were also analyzed through I-Mutant2, Cupsat and ERIS web servers [21–23]. Biophysical characterization was performed using VADAR ver 1.8 web interfaces. Promotif and in-house developed python tool was used for calculating intra-protein interaction. Docking with Triacylglycerol (C8) substrate was performed through Autodock. Wild type and mutated structures were superimposed by using PyMol V0.99. The ranks of the mutated structures and wild type lipases were calculated through RankProt tool and only those mutations, which led to an increase in the rank of the mutated structures with respect to the wild type, were chosen for further *in vitro* validations. Furthermore, a comparative analysis was drawn about difference in thermostability of mutants generated by RankProt and other directed evolution strategies. Four available crystal structures of engineered thermostable mutants of *Bacillus subtilis* lipase obtained by directed evolution (PDB ID: 1t2n, 1t4m, 3d2c, 3qmm).

#### Contact map analysis and molecular dynamics simulations–an insight into the biophysical property of the obtained mutations

The role of the predicted mutations in enhancing thermostability in *Bacillus subtilis* lipases were analyzed by contact map analysis and molecular dynamic simulations. Protein contact map represents the contact distance between amino acid residues in a protein structure using a binary matrix. It has been reported that two residues in a protein are in contact if their Euclidian distance is <8Å [46]. This is presumed to differ from protein to protein and thus can be instrumental in analysis of differences in contacts between mutated thermostable and mesostable *Bacillus subtilis* lipase. Therefore, contact maps of the mutated and wild type structures were constructed using CMView [47]. Combined contact map and 3D structure visualization was performed through PyMol software. As controls of this experiment, comparative analysis of four engineered structures of *Bacillus subtilis* lipase (1t2n, 1t4m, 3d2c, 3qmm) and their wild type structure (1i6w) were also performed. Number of unique contacts in each structure were calculated. Further, HB-plot tool was employed to analyze the network of hydrogen bonds in wild type and the mutated structures [48]. After that MD simulation was carried out using GROMACS-4.5.3 in conjunction with the OPLSA force field for 30ns at 320, 330 and 350K. Secondary structure analysis was performed using the DSSP program. Other analyses such as root-mean-square deviation (RMSD), root-mean-square fluctuation (RMSF), radius of gyration, solvent accessible surface area (SASA), and hydrogen bonds, were performed using tools within the GROMACS simulation package. RMSD calculation was carried out using the starting structure of each simulation as a reference. For hydrogen bond calculations, a donor−acceptor cut off distance of 0.35 nm and acceptor−donor−hydrogen bond angle cutoff of 30° were considered. The visual analysis of structures and preparation of figures was carried out using Pymol and Xmgrace.

#### *In vitro* analysis of the predicted mutations

*Bacillus subtilis* 168-lipase clone in pET21b was a kind gift from Dr. N. Madhusudhan Rao of CCMB, India. The lipase gene of 639bp cloned with the restrictions sites BamHI and NdeI. It was sub-cloned into pET28a vector with the same restriction sites. Multi-site directed mutagenesis was carried out according to the protocol of Agilent Technologies, Quick Change Light multi-site directed mutagenesis kit. Two double mutations were chosen as per ranking given by RankProt. The amino acids in wild type *Bacillus subtilis* lipase (1i6w), T47 and Q121 were mutated to T47S, T47N and Q121N. The primers were 5'-aggcgcttccgggaacagatccaaataataagattttatacacatc-3' for Q121N mutation, 5'-gttgatttttgggacaagacaggcaataattataacaatggaccggtattat-3' for T47N mutation and 5'-ataccggtccattgttataattgctgcctgtcttgtcccaaaaatc-3' for T47S mutation. The mutations were confirmed by sequencing. Plasmids harboring the wild type and two mutant genes (mut 1 and mut2) in pET28a vector were transformed into *E*.*coli* BL21 cells. Transformed cells were induced with IPTG, harvested by centrifugation, washed with STE buffer, resuspended in lysis buffer and sonicated. The sonicated cells were centrifuged at 20,000 rpm at 4°C for 30 minutes and the supernatant was checked for protein expression by SDS- polyacrylamide gel electrophoresis (SDS–PAGE) carried out as described by Laemmli method. Further Ni–NTA purification of the wild type and mutants were performed and finally eluted in 1X TE buffer pH 8 with 250mM imidazole. Fractions showing the presence of protein were pooled and dialysed in 50mM phosphate buffer to remove imidazole. The estimation of protein concentration of the purified fraction was performed in microtitre plate by standard Bradford assay technique using BSA as standard at 595nm [49].

#### Enzyme assay and kinetics of the attained mutations

Lipase was routinely assayed for the wild type and mutants, spectrophotometrically in microtitre plate reader using p-nitrophenyloctanoate (p-NPO) as substrate following the protocol of Glogauer et al. (2011) [50]. One enzyme unit (U) was defined as the lipase activity that liberated 1 μmol equivalent of p-NP ml^-1^min^-1^ under the standard assay conditions. All assays were performed independently and in triplicates. The enzyme activity, specific activity, percentage yield and fold purification was calculated for the wild type and mutant enzymes. The influence of substrate concentration on the reaction velocities of purified lipases was studied with pNPO ester under standard assay conditions. The Michaelis-Menten constant (*K_m_*) and the maximum velocity for the reaction (*V_max_*) were determined from Lineweaver-Burk plot. Enzyme specificity (*K_cat_*) and specificity constant (*K_cat_*/*K_m_*) were also calculated.

#### Determination of optimum temperature for activity and melting temperature (*T_m_*) for stability for wild type and mutant lipases

The temperature giving maximum substrate conversion over a 30 minutes’ reaction time was determined by incubating the wild type and mutant enzymes in a reaction volume of 50μL at various temperature within the range of 30°C to 90°C in Agilent SureCycler 8000 gradient PCR system. Enzyme assay was carried out as described earlier using p-NPO as substrate at 35°C for 10 minutes. All experiments were performed independently and in triplicates. Specific activity and percentage relative activity were calculated and used to compare the thermal stability of the enzymes. Further, the protocol published by Niesen et al. (2007) was followed to measure the melting temperature of the wild type and the mutant lipases. Real-time PCR (Agilent Mx3005p) was used to measure the fluorescence using 200X SYPRO orange dye (Invitrogen). No protein controls having the dye and 50mM phosphate buffer, pH 8 were used for each experiment. The temperatures (*T_m_*) of the transitions for melting curves were calculated from the midpoint of transition of the percentage relative fluorescence intensity (I) of the maximum as a function of temperature (T). The midpoint of transition is the temperature at which 50% of the protein has denatured, and is a measure of the protein's inherent thermal stability [51].

## Results

### Dataset creation and feature selection

As thermostability is relative to mesostability, a binary dataset was created comprising 127 structures of thermostable proteins (TP) and their mesostable homologues (MP) (S1 Table). The data was representation of bacteria (58%) followed by archaea (27%) and eukaryotes (13%). It also included both eukaryotic and prokaryotic proteins belonging to 132 thermostable organisms. For feature generation, numerical data of 25 protein structural features previously reported to contribute to thermostability were calculated. The next step was primary filtering for feature selection by performing two-tail KS test, after which the attributes narrowed down to 17 features (Table 1).

### Ranking features contributing towards protein thermostability

After preliminary feature selection by two tail KS-test, the quest was to find the importance of each feature in contributing towards thermostability. Analytical Hierarchical Process (AHP) was employed as it is a multi criteria decision making approach. The steps have been illustrated in Fig 2A. The ranks have been presented in a scale of 0–1. As seen from Fig 2B, II and MMH occupied the highest rank with priority vector of 0.091. It was interesting to note that IGT, SB and CASA with priority vector of 0.081 were ranked as the second group of major factors contributing towards thermostability.

Notable observations were that total percentage γ-turns (inverse and classic; GT) and hydrogen bonds (HB) in a protein occupied the third rank with priority vector 0.072 and fourth rank with priority vector of 0.071 respectively. Another interesting observation was that total percentage γ-turns (inverse and classic; GT) and hydrogen bonds (HB) in a protein occupied the third rank with priority vector 0.072 and fourth rank with priority vector of 0.071 respectively. This indicates that all type of γ-turns and hydrogen bonds do not equally contribute towards thermostabilizing proteins. This type of priority obtained for the biophysical features indicates that mutations are intricately directed and particular to modulate specific feature space and the global effect may be quite predictable.

Another interesting feature of this study was that AAI, ASI, NASA, PV and PASA occupied lower ranks, which may indicate that increase in such factors have less impact on enhancement of protein thermostability. The next step was finding the accuracy of the method. The CR value is the litmus paper test of AHP method indicative of its accuracy. The CR of the pairwise comparison matrix was calculated to be 0.002. Thus according to Saaty, 2000 the aforementioned judgment to derive at the priority values can be accepted as consistent as the CR value was less than 0.1. pertaining to thermostability [45].

### Development and validation of RankProt

RankProt tool was developed in python as a protein thermostability predictor by using the priority values of the 17 protein biophysical features obtained by AHP for mutation stability calculations. The input to the tool are the mutated and wild type structures of proteins (.pdb format). The mutated structure can be derived using Chimera and stable mutations can be predicted through various available mutation stability prediction tool or designed based on user requirement. The detail of the mutation derivation protocol is explained with *Bacillus subtilis* lipase as a case study in this paper. Another advantage is that multiple mutations can be analysed at the same time. As an output RankProt provides relative ranks for the mutated protein and the wild type. If the rank difference of the mutated protein is positive, then it can be predicted that the mutations performed will lead to enhanced thermostability. It was seen that higher rank difference ensured better thermostability.

RankProt was validated by multi-fold *in silico* blind tests. The first blind test involved 100 thermostable-mesostable pairs and 40 mesostable-mesostable protein pairs randomly chosen from RCSB Protein Data Bank. The dataset used is given in S4 Table and the generated rank difference have been plotted and presented in top left of S1 Fig. The mesostable-mesostable pairs were chosen as a control dataset for reverse validation. The prediction was to see that the relative mean rank differences obtained while ranking thermostable-mesostable pairs are higher (indicating method accuracy) than when mesostable-mesostable pairs are ranked. In line with the prediction, the mean rank value difference obtained for the thermostable-mesostable and mesostable-mesostable protein pairs were 0.09 and 0.01 respectively. This result indicated that RankProt could successfully identify thermostable proteins. The accuracy that we could attain was 91% with Area Under Curve (AUC) of 0.927 calculated from receiver operating characteristic (ROC) curve by fitted maximum likelihood estimation by LABROC4 (Fig 2C). The method for obtaining accuracy has been adopted from Wang et al. 2010 [52]. The result has been presented in Table 2. It can be conclusively said that this method could differentiate between thermostable proteins from their mesostable counterparts with 91% accuracy.

**Table 2:** Validation and accuracy of RankProt.

Blind test set	No. of proteins	Homology	Accuracy%	Mean Rank	Rank Difference
1	100 TP	Homologous	91	0.54	0.09
	100 MP	Homologous		0.45	
2	40 MP	Homologous		0.49	0.01
	40 MP	Homologous		0.50	

As a second method to report the high accuracy in the performance of RankProt, the developed tool was tested on (i) 5 mutated thermostable structures available for *Bacillus subtilis* lipases (retrieved from RCSB PDB) along with its wild type mesostable structure (PDB ID: 1i6w). (ii) 104 mutated structures of bacteriophage T4 lysozyme (mutations leading to gain in stability) with respect to its wild type structure (PDB ID: 2lzm) and (iii) 47 mutated structures of human lysozyme (mutations leading to decrease in stability) with respect to its wild type structure (PDB ID: 1lz1). The third test set is of special importance as the mutants were lower in thermostability w.r.t. the wild type which has a melting temperature of 64.9°C. The results have been presented in S5 Table and S1 Fig. As can be observed from S5 Table RankProt assigned higher ranks to the thermostable mutants, thus positive rank differences were obtained. In another blind test 104 thermostable mutated and wild type structures of bateriophage T4 lysozyme were ranked. From the top right of S1 Fig it can be observed that RankProt performed extremely well in identifying 99 thermostable mutant proteins out of 104 available mutants, with higher rank than the wild type counterpart. In the third test, bottom of S1 Fig, 42 out of 47 loss of function mutants were given lower ranks, thus negative rank difference was obtained. Hence it can be inferred that RankProt was successful to assign higher ranks to the thermostable mutants. It may be noted here that as the ranks are relative the obtained the rank value of the mesostable counterpart differs from case to case and is not a constant. Thus to identify thermostabilising mutations, the observable value should be the rank value difference between the proteins being compared. Therefore, it can be reported here that the method was successful in identifying multiple stabilizing mutations and predict the consequence of mutation on the protein three-dimensional structures.

#### Case study: Prediction and ranking mutations for thermostabilizing *Bacillus subtilis* mesostable lipase

To show the practical applicability of RankProt *Bacillus subtilis* lipase was chosen as a case study. 20 single point mutations were predicted to be stabilizing by the available fourteen mutational stability prediction servers for *Bacillus subtilis* lipase and the obtained mutations were ranked by RankProt relative to the mesostable structure (1i6w). Out of these 20 single point mutations, 18 combinations of double mutations were ranked higher than the mesostable counterpart. Further, the two double mutations that resulted in highest and lowest rank value difference were chosen for further analysis. The mutations were in residues T47S, Q121N (mut 1) which had the rank of 0.5 relative to the wild type with rank 0.49. T47N, Q121N (mut 2) had the rank of 0.54 relative to the wild type with rank 0.45. The rank difference of mut 2 with that of the wild type (rank difference of 0.09) was greater than mut 1 (rank difference = 0.01).

#### *In silico* validation

Analysis of biophysical properties of wild type and mutated proteins showed that there was increment in γ- turns (GT), salt bridges (SB), ionic interaction (II), cation-π interaction (CPI), non- polar accessible surface area (NASA), main-chain side-chain (MSH) and side-chain side-chain (SSH) hydrogen bonds in mut 1. In mut 2, there was increase in salt bridge (SB), γ-turns (GT and IGT), charged accessible surface area (CASA), hydrophobic interaction (HI), main-chain main-chain (MMH), main-chain side-chain (MSH) and side-chain side-chain (SSH) hydrogen bonds. The reason behind the obtained lower rank difference for mut 1 can be attributed to the decrease in main-chain to main- chain hydrogen bonds (MMH) and charged accessible surface area (CASA) which were ranked as high priority thermostability predictors. This result led to the prediction that mut 2 will be more thermostable than mut 1. Further, molecular superimposition in PyMol showed low RMSD values of 0.278 (mut 1 and 1i6w) and 0.312 (mut 2 and 1i6w), indicating that the wild type and mutated structures were similar without any massive structural changes. Analysis of the effect on activity of the mutants by molecular docking with (C8) substrate, resulted in binding energy of -4.49 (mut 1), -4.91 (mut 2) and -5.53 (1i6w) for the mutated and the native structures respectively. This showed that the mutations did not affect the catalytic pocket of the lipase.

**Fig 3:**
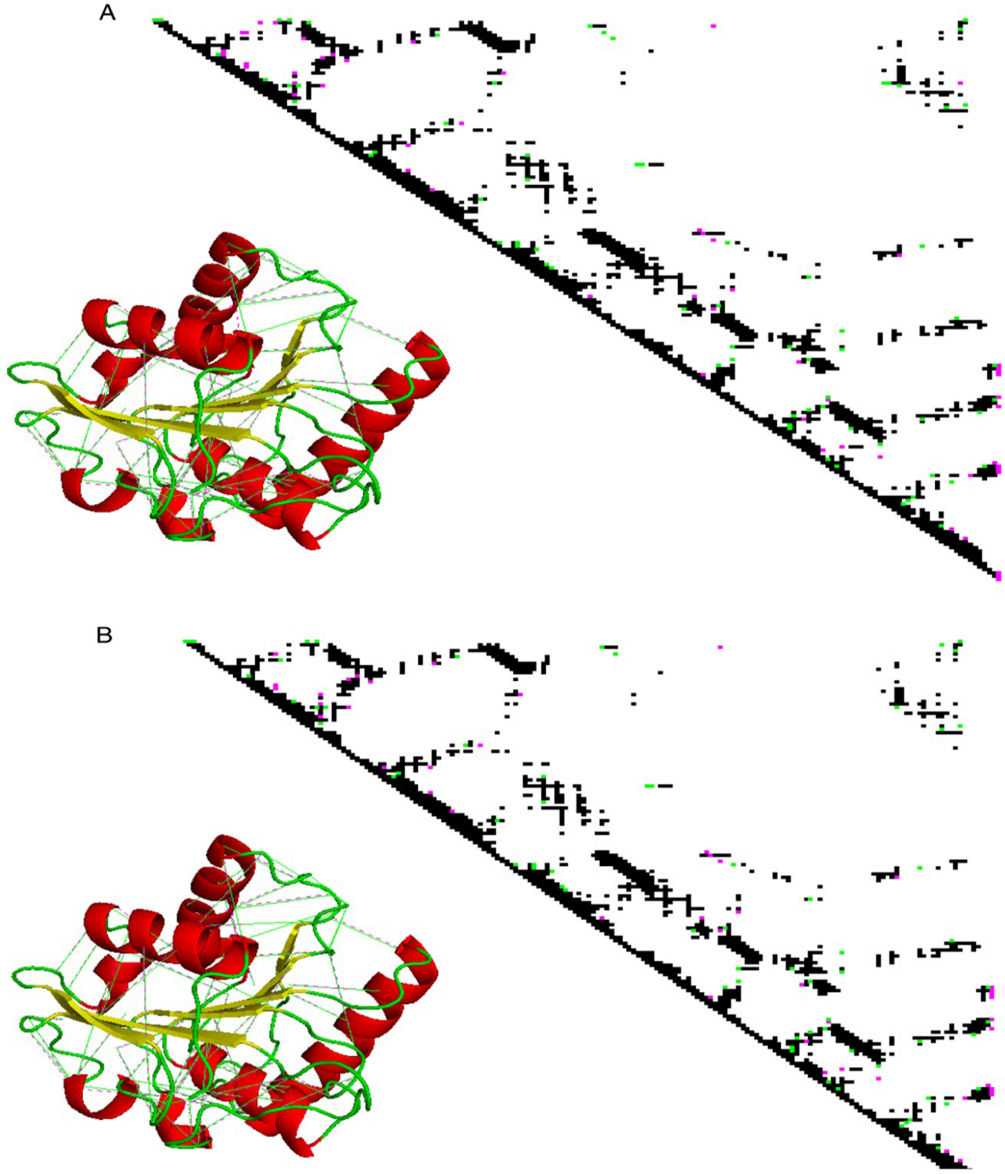
Graphical illustration of unique contacts formed in A mut 1 vs. 1i6w along with the cartoon representation of mut 1 showing its unique contact. B mut 2 vs. 1i6w along with the cartoon representation of mut 2 showing its unique contact.

**Fig 4:**
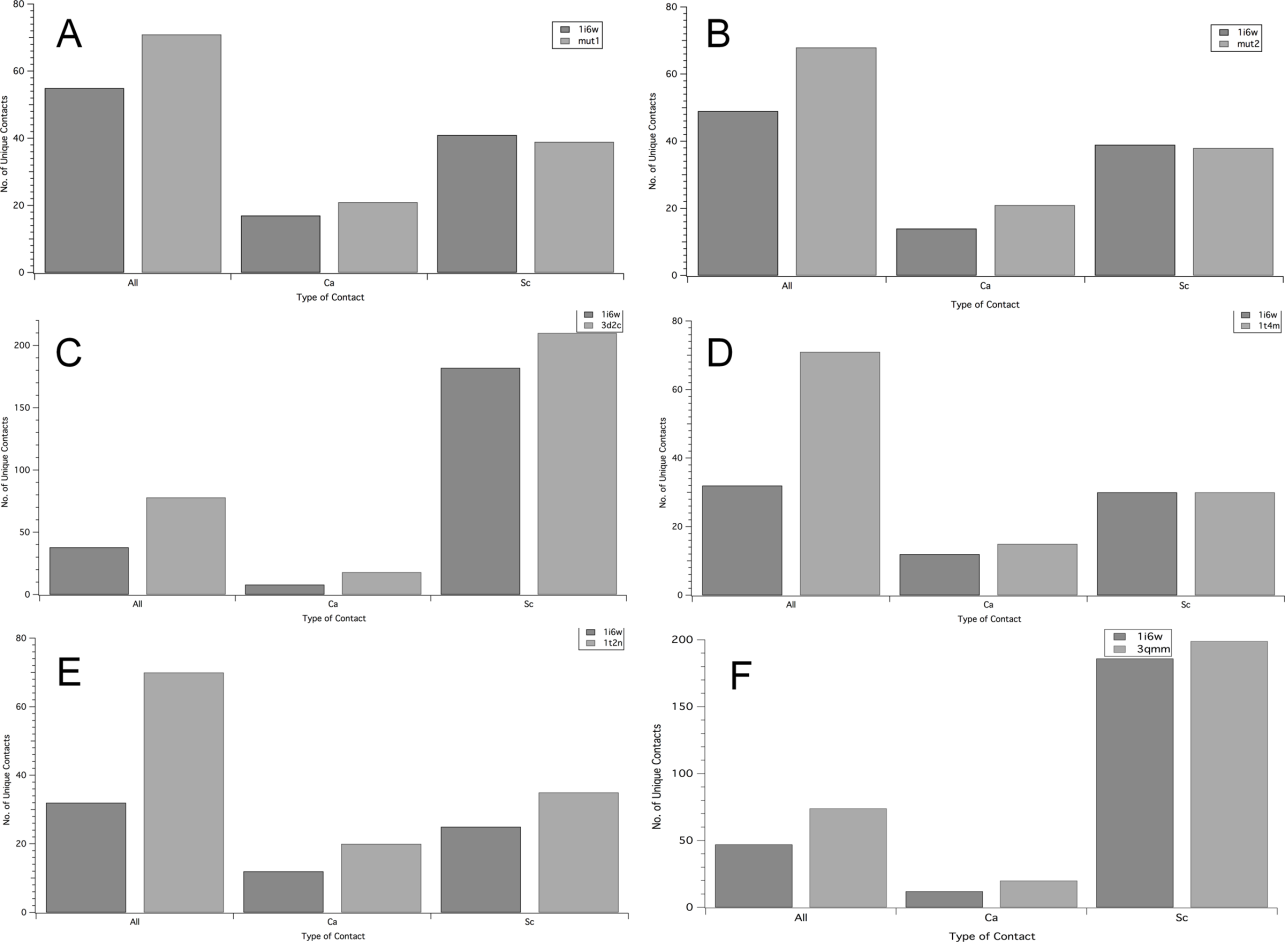
Comparative bar graphs of unique contacts in thermostable mutants of Bacillus subtilis lipase and wild type (1i6w). A 1i6w vs. 1t2n; B 1i6w vs. 1t4n; C 1i6w vs. 3d2c; D 1i6w vs. 3qmm; E 1i6w vs. mut 1; F 1i6w vs. mut 2. All represents all unique contacts in PDB structure, ca represents backbone unique contacts and sc represents side chain unique contacts.

To further analyse whether our prediction was accurate regarding the higher thermostability of mut 2 than mut 1 due to higher rank difference of mut2 compared to mut1, intra-protein contact map analysis of the mutants w.r.t. the wild type was performed. Unique contacts in mut 1 and mut 2 in comparison to the wild type (PDB ID: 1i6w) have been illustrated in Fig 3. It was calculated that number of unique contacts in mutants were much higher than the wild type structure. Furthermore, superimposition of wild type and mutant structures with their unique contacts revealed that the unique contacts were more in the loop region of the 3D-structure. Analysis of the total number of unique contacts, as illustrated graphically in Fig 4, for all the *Bacillus subtilis* thermostable mutants available in RCSB PDB were observed to be greater than the wild type. Backbone unique contacts also increased in the mutants. Through comparison of four available crystallized thermostable structures of *Bacillus subtilis* lipases in PDB, it was further observed that 1t4m and mut 1 did not show any increase in unique contacts pertaining to their side chains. Also, side chain unique contacts were lower for mut 2 and were more for 1t2n, 3d2c and 3qmm. The total number of contacts formed was also higher than the number of contacts lost, suggesting an increase in the compactness of the protein. Similar inferences were drawn by Srivastava et al. 2014 when they performed network analysis of thermostable mutants of *Bacillus subtilis* lipases showing gain of contacts leading to “regional stability” in the loops and termini of the structures. Thus it could be inferred here that mut 2 had high probability of being thermostable.

The analysis of hydrogen bonding network in wild type and mutated structures of *Bacillus subtilis* lipase uncovered that main-chain main-chain hydrogen bonds increased in four mutated structures (3d2c, 1t4m, 3qmm, mut 1 and mut 2). Main-chain side-chain and side-chain side chain hydrogen bonds increased in all the mutants (S2 Fig). Along with increase in the number of hydrogen bonds it was observed that hydrogen bonds <3Å were much greater for the mutants in comparison to the wild type structures (S3 Fig). Therefore, it can be concluded that as the temperature stability increases, number of short distance hydrogen bond also increases. It was also observed that intra-molecular hydrogen bonding networks increased near the β-strand and α-helices of the mutants. Therefore, it can be concluded that as the temperature stability increases, number of short distance hydrogen bond also increases.

**Fig 5:**
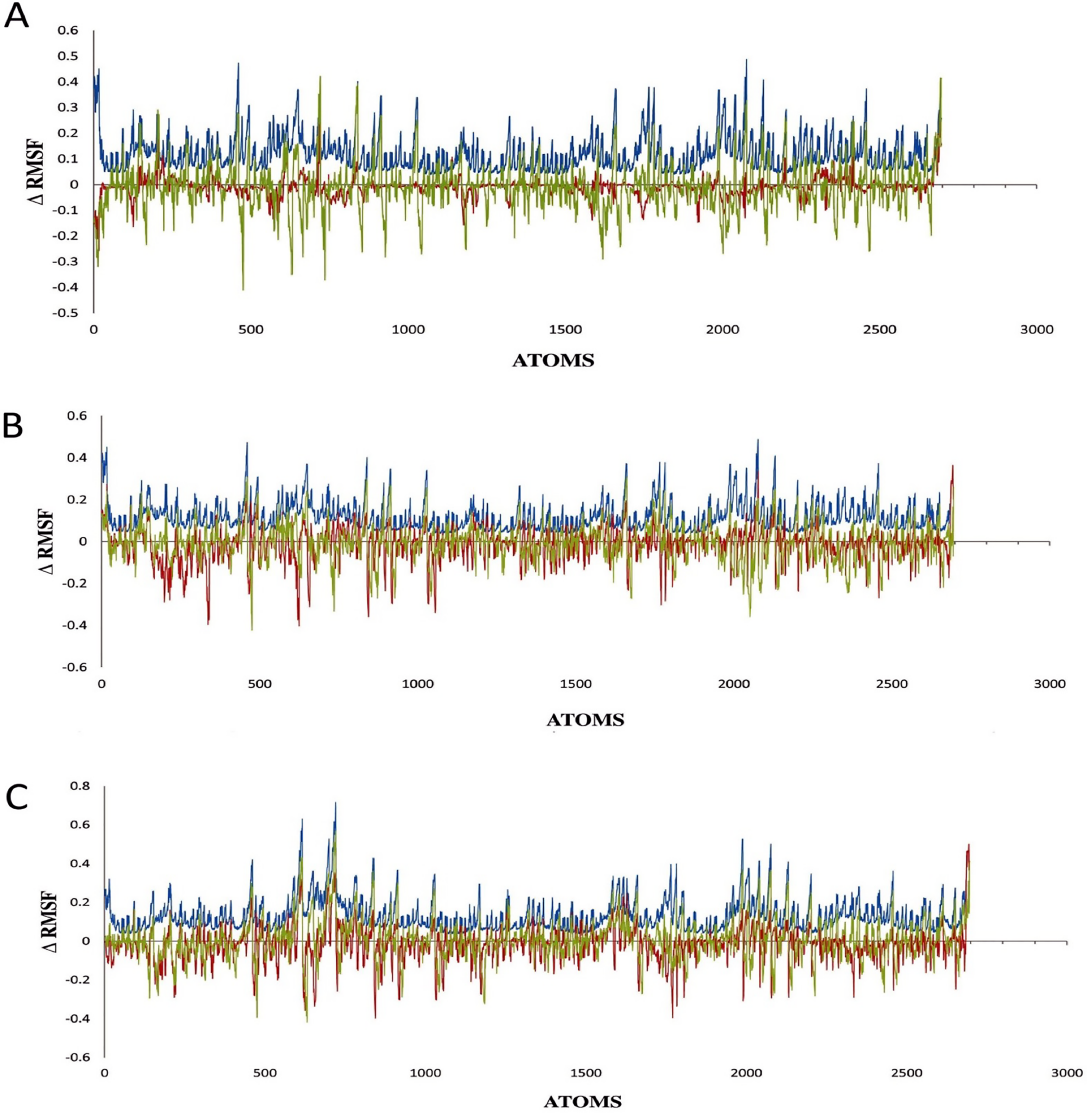
Graph showing difference in RMSF between mut 1, mut 2 and wild type lipase at A 320K, B 330K, C 350K.

MD simulation at higher temperatures for wild type (1i6w: WT), mut 1 and mut 2 were also performed to analyse the stability of the mutated structures with respect to the rank value differences they were associated with. RMSD, RMSF, Radius of gyration, hydrogen bonds and secondary structure analysis of the trajectory, were performed after 30 ns MD simulation at three different temperatures (320K, 330K and 350K). The global average RMSD was similar for mut 1 and wild type at all temperatures. But at higher temperature of 350K the RMSD of mut 2 was much lower than mut 1 and wild type. Thus it was assumed that mut 2 was more thermostable than mut 1 and the wild type. Average RMSF plot showed global reduction in flexibility for mut 2 at 350K while mut 1 shows an increase. Appreciable difference of RMSF between wild type and mutants was observed at the C- terminus. Thus it could be inferred that the C-terminus of *Bacillus subtilis* lipase played important role in its stability. At 320K (S4 Fig) the C-terminus was more stable in mut 1 than mut 2. At 330K (S5 Fig), both N- and C-terminus were less flexible than wild type. This showed that the predicted mutations by RankProt resulted in lowering of flexibility of the mutants. At 350K the flexibility of the wild type and mutants, arranged in descending order was mut 2> mut1>wild type (S6 Fig). Again, the average value of RMSF showed that mut 2 had considerable reduction in the RMSF values globally. The difference plots of RMSF of mut 1 (Red lines) and mut 2 (blue lines) with wild type (green lines) at 320K, 330K and 350K has been illustrated in Fig 5. The comparison highlighted that the reduction in the flexibility was observed in regions where mutations were performed which were specifically at the atom numbers 689–702 for T47 and 1783 to 1796 for Q121 in mut 1 and mut 2.

**Fig 6:**
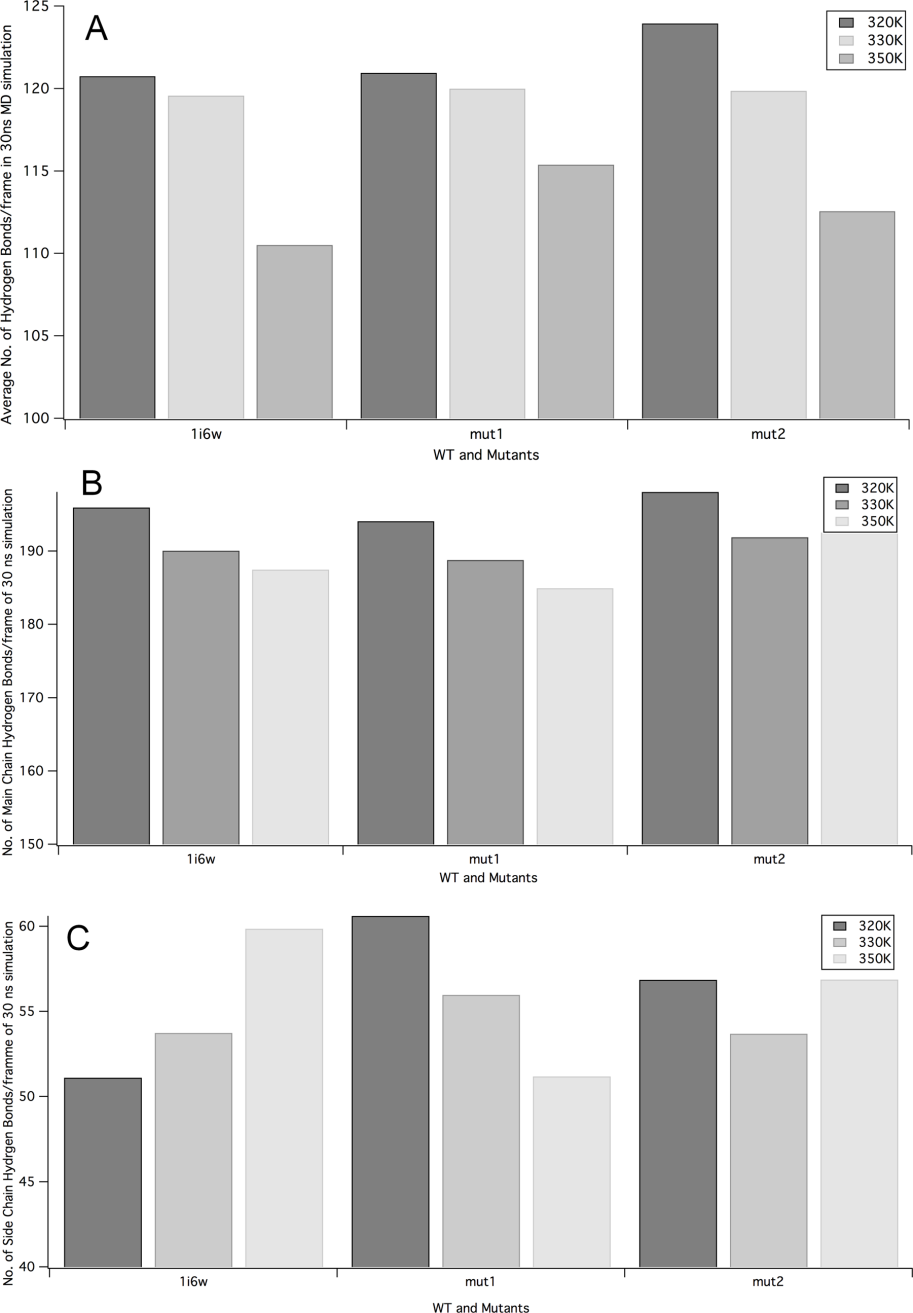
The average number of hydrogen bonds per frame of the 30 ns MD simulations A All hydrogen bonds. B Main chain. C Side chain hydrogen bonds at 320, 330 and 350K.

Further, the radius of gyration was analysed in a time-dependent manner to investigate the compactness of wild type and mutants. Lower average Rg of mutants at all the three temperatures reflected that the mutants were more compact than the wild type. The radius of gyration reflects on the packing of amino acids throughout the simulation thereby stability and folding rate. At 320K mut2 was more compact than mut 1 and wild type. However, at 330K mut 1 becomes more compact than mut 2 but the compactness of mut 2 was still lower than the wild type. Further, at 350K mut 2 was more compact. Therefore, mut 2 was considered more stable than mut 1 and wild type at elevated temperatures. Analysing the average number of hydrogen bond changes in the trajectory revealed that the average number of intra-protein hydrogen bonds was much higher for mut 2 throughout the simulation at different temperatures (Fig 6). Interestingly average number of main chain hydrogen bonds was also much higher for mut 2 followed by wild type and the least was observed for mut 1. The highest prioritized criteria by RankProt are main chain hydrogen bonds. Therefore, proteins which show an increase in such bonds will be more thermostable. It can be estimated that mut 2 will be more stable than mut 1. Analysing the secondary structure changes in mut1 and mut2 showed that at all three simulated temperatures, the percentage regular secondary structures did not undergo drastic alterations (S6 Table). It was also interesting to observe that residues forming the turns and beta sheets at higher temperature were more stable in mut 2 than mut 1. This shows that mutants were more stable than the wild type at higher temperatures.

#### *In vitro* validation

The wild type lipase was successfully cloned into pET28a vector with N-terminal His tag. Mutations at Q121N, T47S (mut 1) and Q121N, T47N (mut 2) performed through site directed mutagenesis was successful as observed through sequencing results. S7 Fig is the gel electrophoresis picture after restriction digestion of the wild type and mutants through NdeI and BamHI. Wild type and mutant proteins gave bands on a stained SDS-PAGE gel which is corresponding to 23KDa (S8 Fig). The purity of lipase after affinity purification increased by 10.8, 2.9 and 4.7 folds over the crude extracts of wild type, mut 1 and mut 2 lipases respectively. The percentage yield was calculated to be 84%, 74% and 79% for wild type, mut 1 and mut 2 lipases respectively. The specific activities of wild type and mutant lipases on hydrolysis of p-nitrophenyloctanoate were observed to increase after affinity purification. Kinetic parameters for *K_m_*, *K_cat_* and (*K_cat_*/*K_m_*) were calculated (Table 3). It was observed that mut 1 did not show an enhancement in activity (*K_cat_*/*K_m_*). mut 2 showed 3-fold enhancement in activity. From Table 3, mut 2 can be called catalytically more efficient with a lower *K_m_* value than wild type. However, the *K_m_* value increased for mut 1. Therefore, it can be concluded that mut 2 was the most catalytically efficient lipase among mut 1 and wild type.

**Table 3:** Enzyme kinetics parameters of the wild type and mutant lipases.

Lipase	*K_m_* (mM)*	*K_cat_* × 10^3^ (*min*^−1^)	*K_cat_*/*K_m_* × 10^3^ (*mM*^−1^*min*^−1^)
WT	0.64 ± 0.85	2.208 ± 1.28	3.450
mut 1	1.33 ± 1.24	1.399 ± 0.97	1.051
mut 2	0.44 ± 1.53	4.280 ± 0.43	9.727

*Kinetic parameters were estimated from assays conducted at 35°C using PNPO as substrate.

#### Temperature stability of the thermostable mutants

**Fig 7:**
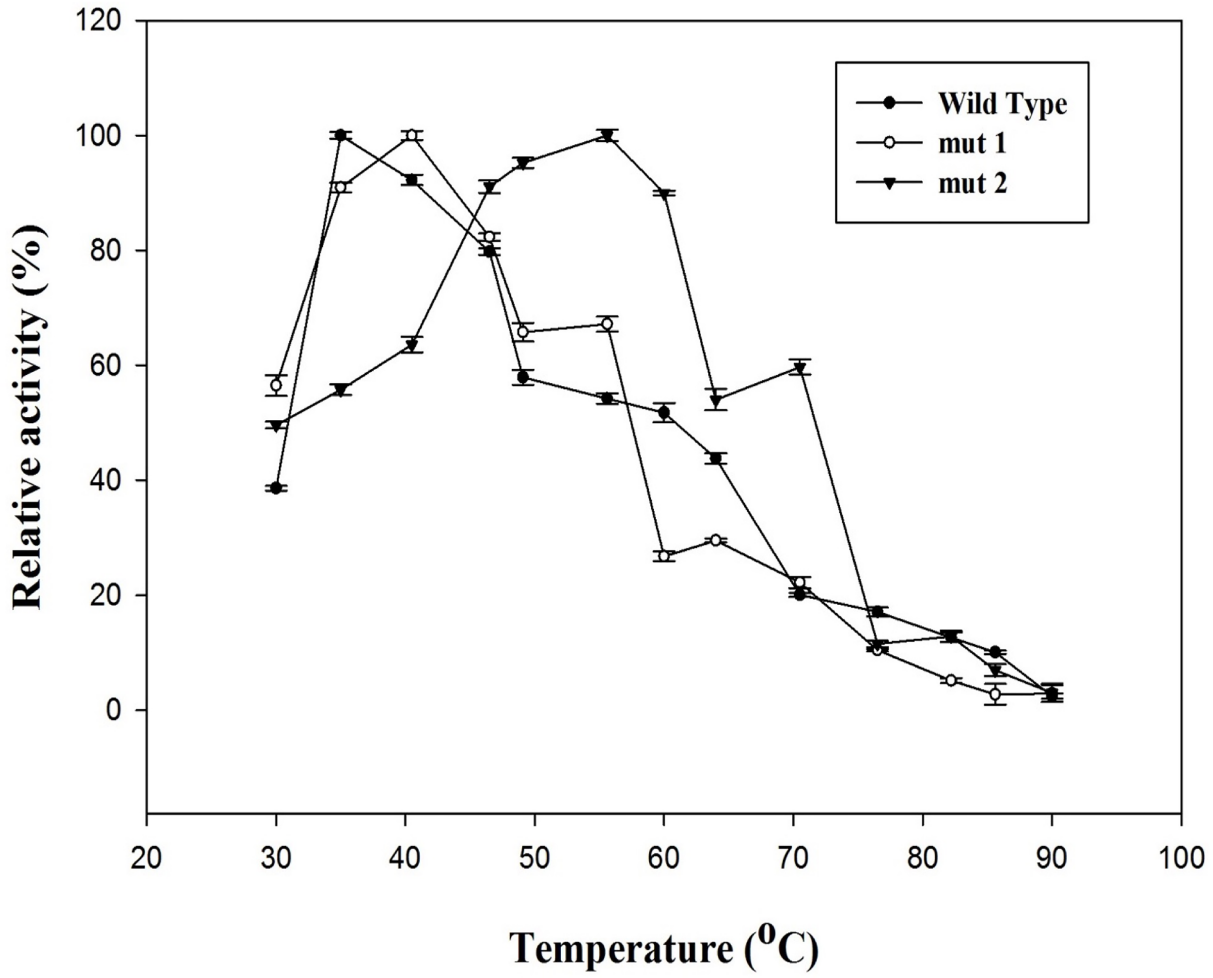
Graph showing % relative activity of wild type, mut 1 and mut 2 lipases from temperatures 30–90oC. Error bars are the standard deviations of experiments done independently and in triplicates.

The temperature effects catalyzed by wild type and mutant lipases from *Bacillus subtilis* has been illustrated in Fig 7 for different temperature range at pH 8.0. The enzyme relative activity for wild type lipase was maximum at 35°C, being stable with temperature in the range of 30°C– 50°C, with a sharp decrease at temperatures above 50°C with only 20% of activity left at 60°C. It was observed that mut 1 retained optimum activity in between 30°C to 45°C; attaining maximum activity at 40°C and the activity dropped slightly thereafter. The temperature stability of mut 2 was observed to increase from 30–55°C and the activity dropped thereafter. The maximum activity was observed at 55°C. Comparison of the temperature stability of mut 1 and mut 2 with previously reported thermostable mutants of *Bacillus subtilis* lipase obtained by directed evolution strategies (Table 4) showed that this represents the fewest number of predicted mutations that resulted in enhancement in thermostability by 20°C.

**Table 4:** Temperature stability of *Bacillus subtilis* lipase mutant crystal structures obtained from PDB generated by directed evolution.

PDB ID	No. of mutation	mutation: location	*T_m_*°C	*T_opt_*°C
1i6w (WT)	0	None (wild type)	56	35
1t2n	3	L114P: loop	61.2	45
3d2a	4	I157M: 310 helix	63.4	N/A*
3d2b	6	F17S: 310 helix, N89Y: C-terminal helix	67.4	50
3d2c	9	A15S: loop, A20E: N-terminal helix	71.2	55
3qmm	12	M137P: loop, S163P: N-terminal helix	78.2	65
Mut 1	2	T47S:loop, Q121N: loop	63	40
Mut 2	2	T47N: loop, Q121N: loop	66	55

*N/A: Not available

**Fig 8:**
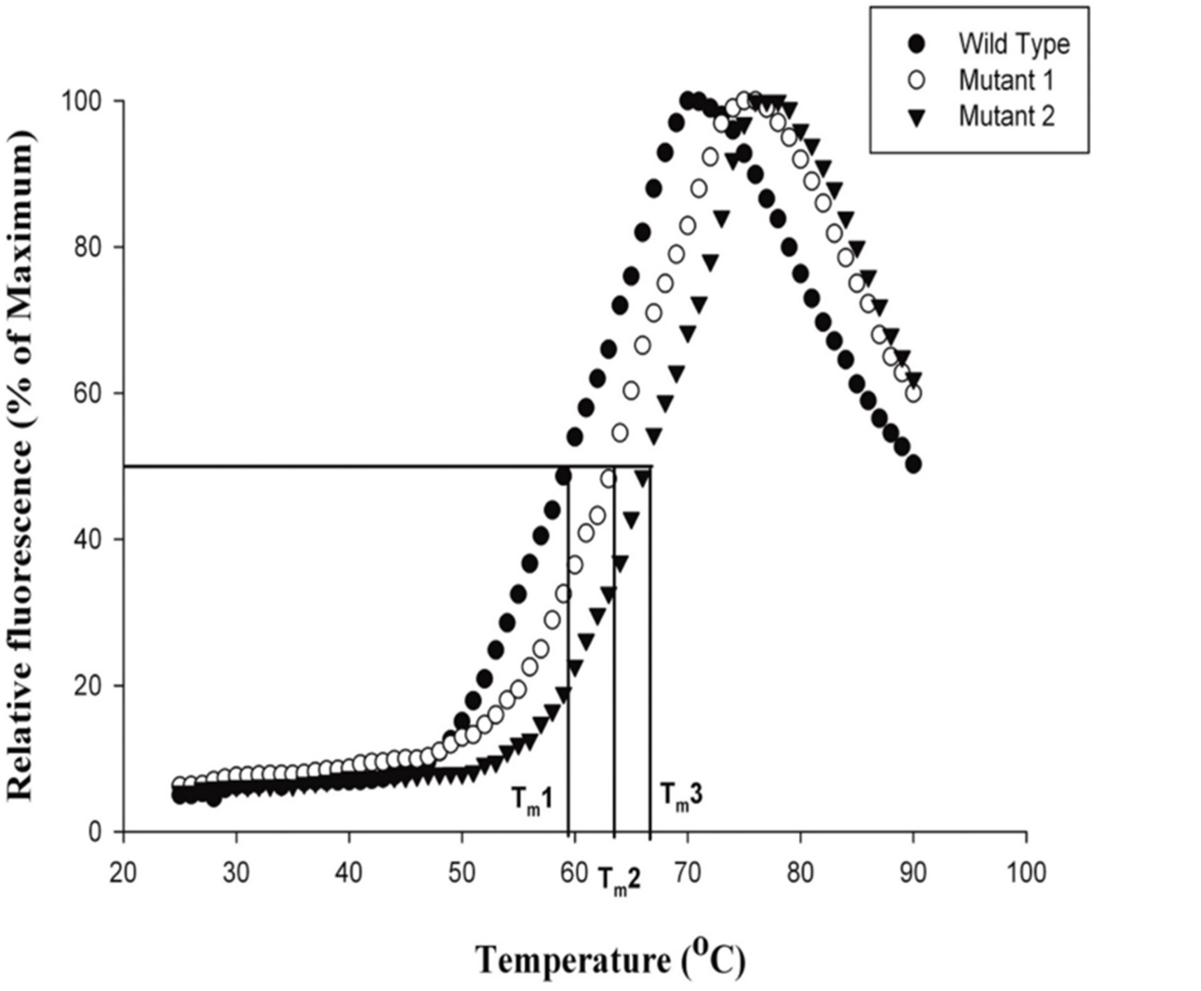
Thermostability graph of wild type and mutant lipases. W for wild type lipase; M1 for mut1 and M2 for mut 2 lipases. The mid-point of fluorescence transition or where 50% relative fluorescence is achieved is the T_m of the enzymes. The calculated T_m for wild type, mut 1 and mut 2 were: T_m1: 59oC, T_m2: 63oC, T_m3: 66oC.

Calculating the melting temperature (*T_m_*) by Thermofluor technique resulted in thermal profiles of wild type, mut 1 and mut 2 as illustrated in Fig 8. The T_m_ of the mutants was calculated to be 63°Cand 66°C with respect to the wild type which has a *T_m_* of 59°C. Therefore, mut 2 was more thermostable than mut 1. Further, the *T_m_* of the mut 1 and mut 2 were comparable to those of previously reported thermostable mutants of *Bacillus subtilis* lipase; 3d2a and 3d2b respectively (Table 4) with 4 and 6 mutations induced randomly through error prone PCR. It can be concluded here that though both the mutants possessed increased temperature stability than the wild type, the thermostability of mut 2 was greater than mut 1 because the rank of mut 2 was greater than 0.54 which was in-turn greater than mut 1. Moreover, contact map and MD simulation analysis also predicted mut 2 to have greater stability than mut 1. Therefore, the methodology employed for attaining thermostable *Bacillus subtilis* mutants by ranking of mutations through RankProt was successfully validated.

## Discussions

A complete understanding of the rationale behind protein thermostability and to use this understanding to develop thermostable proteins in laboratories will require more investigations in the future. We have however made some progress towards this goal. This work showed that features related to intra-protein non-covalent interactions (biophysical interactions) can be used to select multiple mutations that will enhance protein thermostability. This was achieved by enumerating the intra-protein biophysical interactions as features from a binomial protein dataset comprising of 127 thermostable and mesostable homologous protein structures. Restricting the binomial dataset only to protein three dimensional structures was taken as a mandate as it is known that function of a protein is determined by the conformational arrangement of amino acid residues in the three dimensional structure space. As thermostability is said to be independent of phylogenetic diversity, the dataset consisted of proteins from all phylogenetic groups [53]. Further, the dataset covered all six enzyme classes and structural proteins. Therefore, the robustness and unbiased nature of the dataset can be inferred which makes the platform set for further research on protein thermostability.

This binomial dataset was used to calculate features related to biophysical interactions from the three dimensional structures of the homologous pairs of thermostble-mesostable proteins. Most of the features selected in this work have been previously mentioned to be important in enhancing protein stability by various researchers [2, 54–55]. As a new member in the feature list- γ-turns were introduced. Such turns have been reported to be an important feature for protein thermostability in lipases in one of our earlier work [55]. It can be noted here that features and their subtypes were considered together due to the belief that in contributing towards certain functional properties of any protein, the overall contribution of certain features is the cumulative effect of the contributions of its sub-types. Individually they may show positive or negative effect but globally their effects can balance out- in comparison to their mesostable counterparts, resulting in a subtler effect. Good examples can be found in the earlier works [2, 54–57]. This can be observed for the feature of total percentage of hydrogen bonds and its sub-types (main-chain to main-chain, main-chain to side-chain and side-chain to side-chain) and total percentage of γ- turns with its sub-types (classic and inverse).

The originality of this work was in using multiple biophysical interactions as feature predictors of protein thermostability and ranking these features by employing AHP in accordance to their importance in contributing towards protein thermostability. AHP has been previously used in industrial engineering applications, selecting ecological indicators for river flow restoration, and in health care research. As per our knowledge, it is being employed for the first time to solve multi criteria decision making problem for predicting protein thermostabilizing mutations. [43–44]. After applying AHP for feature ranking and obtaining their corresponding eigen values, it was interesting to observe that the features were clustered to ranked- groups and each group corresponded to equal priority of these features in contribution towards thermostability. The highest ranked group of features as predictors of protein thermostability consisted of ionic interactions and main-chain to main-chain hydrogen bonds. Previous research reported ionic interactions and main chain hydrogen bonds as important factors contributing towards protein thermostability [58–59]. For example, Vetriani et al. (1998) observed reduction in inter subunit ion-pair network in the less stable mutated glutamate dehydrogenases from *Thermococcus litoralis*. Restoring the interactions resulted in enhancement in thermostability at 104°C in comparison to the wild type [59]. Again, ample literature exists on the enhancement of thermostability due to increase in hydrogen bond. For example, Vogt et al. (1997) examined 16 protein families of thermophilic and mesophilic proteins and concluded that increase in number of hydrogen bond enhances protein thermostability [60]. Among all the types of hydrogen bonds, main-chain to main-chain hydrogen bonds can be important in thermostabilizing proteins as they are considered to have lower configurational entropy [20]. Further, Sadegi et al. concluded main-chain hydrogen bonds to be an important factor contributing towards thermostability by drawing an elaborate analysis on a dataset containing 60 structures of thermophilic proteins and their mesophilic homologues [54].

The next high priority ranked feature group included inverse γ- turns, salt bridges and charged accessible surface area. In γ- turns, hydrogen bond forms between one main chain carbonyl oxygen to the main chain N-H group two residues along the chain. Such hydrogen bonds are also known as short strong hydrogen bonds with a distance <2.7Å and have been previously reported to increase thermostability of lipases [56]. It is also worth mentioning here that these results corroborate our previous attempts to relate thermostabilizing features at all the hierarchies of protein organization through machine learning methodologies which revealed that main chain hydrogen bonds, inverse γ-turns and aromatic interactions were important to enhance protein thermostability [57]. Further, the third and fourth ranks consisted of the total percentage γ-turns (inverse and classic γ-turns) and total hydrogen bonds in a protein respectively. This indicates that all type of γ-turns and hydrogen bonds do not equally contribute towards thermostabilizing proteins.

It was further interesting to note that aromatic and polar interactions occupied lower ranks, indicating that increase in such features may have less impact on enhancement of protein thermostability. Findings on enhancement in stability due to increase in aromatic interactions exist [20, 60]. It has been reported that aromatic interaction improved the thermostability of mesophilic xylanase [20]. But, literature ceases to exist covering the spectrum of all six enzyme classes where increase in aromatic interaction have been studied for thermophilicity enhancement. Results of Kumar et al. who examined 18 non-redundant families of thermophilic and mesophilic proteins, reported that consistent pattern of differences in contribution of packing volume, polar and non-polar surface area exist between mesophilic and thermophilic proteins [61]. This corroborates with the low priority obtained for such factor in this study. Karshikoff et al. (1998) have also reached to similar conclusion about the decrease in packing volume in thermostable proteins [62].

To proceed further with the work of identifying thermostabilising mutations, the calculated priority values of features were employed to write a thermostabilising mutation prediction tool- RankProt. It is based on the AHP approach discussed earlier, trained by ranks of protein's biophysical interactions as features, and uses these values to predict thermostabilizing mutations. The existing state-of-the-art tools to predict stability changes on point mutations in chronological order are FoldX (3.0, beta 6.1) [20], I-Mutant [21], CUPSAT [23], ERIS [22], PoPMuSiC [25], SDM [26], STRUM [27], EASE-MM [28], HoTMuSiC [30], T-MP-2 [29]. These stability prediction algorithms are mostly knowledge based, few are machine learning based and further lesser are based on molecular dynamics [*T_m_* (melting temperature) changes upon mostly single site mutations except ERIS (predicts changes on single and multi-site mutations) [22]. It can be also said here that predicting accuracy of the existing methods is also restricted by the availability of measured and well documented ΔΔG of proteins and by the training datasets. On the other hand, the outputs of RankProt are relative ranks of the mutated and wild type protein structures. Thus, comparing the AUC (0.927) obtained by RankProt with other existing tools that predicts thermodynamics or melting temperature changes as outputs was not justified. Further, restriction due to the unavailability of the source code of these tools, we could not compare our datasets performance accuracy with that of the available tools. It can be reported here that RankProt is advantageous than the ones explained above as it is also capable of enumerating the effect of multiple mutations. This relates to the probability of higher success rate of attaining *in vitro* thermostable mutants, as multi-point mutations were predicted to have >50% success rate [9]. The other concern is that unlike RankProt, the available algorithms do not provide the details about the biophysical interaction rearrangements occurring due to the mutations. Thus, RankProt performs better in all these contexts being simple in execution and depending on relative ranks of mutated and wild type structures rather than being dependent on thermodynamics or melting temperature changes. Hence, the present developed method would be highly instrumental in the area of recombinant production of thermostable proteins in the shortest possible time.

Regarding the practical applicability of RankProt it can be said that, RankProt passed all *in silico* validation tests with AUC 0.927. It could classify thermostable mutants (*Bacillus subtilis* lipases, bacteriophage T4, Human lysozyme) by providing them with higher ranks relative to their non-mutated wild type. Thus RankProt was further used to evolve thermostable *Bacillus subtilis* mesostable lipase by predicting thermostabilizing mutations. Mutations were predicted for *Bacillus subtilis* mesostable lipase and two double mutants were developed *in vitro* (mut 1 and mut 2; mut 2 had higher stability rank difference with the wild type structure than mut 1). The biophysical interactions which showed increase in the mutant lipases (*in silico* studies) relative to the wild type, have been previously implicated to increase thermostability of proteins by various researchers [2, 54–55]. The results suggest that the mutants were more compact than the wild type structure. It was interesting to note that packing volume decreased in both the mutants. Compactness has been earlier proposed to enhance temperature stabilily by increasing protein rigidity [63]. Analysis of the secondary structure of wild type and mutants further uncovered that new inverse γ-turns formed in the mutants, near helix 7. Such turns may be involved in stabilization of the helices by formation of intra-molecular hydrogen bonds. Hydrogen bonds were reported to be dominantly involved to achieve temperature stability. Further, main chain and side chain hydrogen bonds have been previously linked to protein thermostability by various researchers [2, 54]. It was also observed that intramolecular hydrogen bonding networks increased near the β-strand and α-helices of the mutated lipases. This can result in better packing due to pinning of helices and stands rendering them rigid to unfolding at elevated temperatures. These aforementioned results substantiate previous findings that increment in hydrogen bonds leads to thermostabilization of proteins [55]. Increase in short distance hydrogen bonds can lead to better stability of proteins as it was reported that as distance becomes smaller, the charge transfer contribution to the hydrogen-bond energy increases and the angle decreases [64].

Further, extensive analysis by performing contact map analysis, molecular docking and 30 ns MD simulation for the two mutants and the wild type structures, revealed that the predicted mutations were stabilizing. RMSD plots of the thermostable mutants were comparable with the work by Huang et al. who reported low RMSD value of thermostable mutant of Cocaine esterase and Singh et al. (2015) who showed that RMSD plots of the thermostable mutants obtained by directed evolution (PDB ID: 1t4n, 1t2n, 3d2a, 3d2b, 3d2c) were lower than that of the wild type *Bacillus subtilis* [65–66]. RMSF analysis of the simulated trajectories showed lowered flexibility in the N- and C- terminal of the mutants. It was earlier reported that docking of N- and C- terminus resulted in reduction of flexibility and thus was responsible for enhancing protein stability [2, 65–67]. Therefore, the obtained results corroborated previous work and suggests that reduced flexibility of N-terminal and C-terminal of mut 1 and mut 2 can play crucial role in their temperature stability. Number of hydrogen bonds were also enhanced in the simulation trajectories of the mutants. This corroborates with results of Srivastava et al. (2014) who by performing MD simulation found that hydrogen bonds increase in thermostable mutants of *Bacillus subtilis* lipase [68]. This endorses our finding that AHP prioritized main chain to main chain hydrogen bonds to be a major contributor for increasing protein thermostability. It has also been previously reported that total number of residues occurring in regular secondary structure is an indicator of the stability of a protein [68]. This corroborates with the observation that at all three simulated temperatures, the percentage of regular secondary structures and average number of residues forming these secondary structures were higher in mutants compared to the wild type. β-sheets were also observed to be enriched in the mutants. This corroborates with results obtained by Leuenberger et al. (2017) who reported enrichment of beta sheet structures in thermostable proteins [69]. Notably, it was observed earlier that mutants have higher number of γ- turns in the loop region than the wild type. Therefore, it adds strength to our earlier statement that γ- turns may increase the stability of the mutants by stabilizing the turns.

As the *in silico* experiments showed that the mutations were thermostabilising, the predicted mutations were then incorporated by performing multi-site directed mutations on the wild type clone of *Bacillus subtilis* lipase. Protein purification, enzyme assay of the purified mutated and wild type lipases showed enhancement in thermostability and kinetic stability. Such type of enhancement in the kinetic stability of *Bacillus subtilis* mutants engineered by directed evolution was observed by Acharya et al. (2004) [70]. The stability of mut2 (stability rank difference 0.09) was higher than mut 1 (stability rank difference 0.01). This corroborates with our results obtained by *in silico* studies. It can be inferred here that the present method not only enhanced the thermostability of mutants but also had a positive effect on their catalytic efficiency. Conclusively it can be said that RankProt can be employed for prediction of thermostabilizing mutations and generation of thermostable mutants in laboratory. It can be reported here that this is the minimum number of mutation in *Bacillus subtilis* lipase that led to such an increase in temperature stability as compared to those thermostable mutants previously obtained by directed evolution techniques.

## Conclusions

In this article, we presented a significant progress toward the design thermostable mutants. The novelty of this work was in using a multi criteria decision making method (AHP) in developing a tool to predict protein thermostabilising mutations based on prioritizing protein biophysical interaction as features as contributors of thermostability. The different priority groups comprising of combinations of various biophysical features related their importance in contribution towards protein thermostability. The ranks obtained for the features also revealed that mutations can be intricately directed by modulating the biophysical feature space leading to increase in protein thermostability. The relevance of this work is that by employing the developed tool, circumventing the requirement of selection pressure and colony screening to obtain thermostable mutants was made possible. It is more robust than the existing tools that predict protein thermostability changes upon mutations, being independent of predicting thermodynamic or melting temperatures changes which is a challenge in itself requiring complicated calculations, computational powers and proficiencies. Additionally, this tool can not only be applicable to predict thermostabilising mutations of enzymes, but also can be used for predicting thermostabilising mutations for any non enzymatic or structural proteins. Further, being able to predict the effect of multiple mutations is an added advantage of RankProt in achieving higher probabilities of success for in vitro evolution of recombinant thermostable proteins. This ensures results in a shorter possible time by utilization of lesser effort and capital in comparison to the random approaches of directed evolution. In future, the rationality of this method can be further tested on other selection pressures that enhance protein stability in extreme conditions.

## Supporting information

S1 Table The 127 thermostable and mesostable protein pairs forming the final dataset. (PDF)

S2 Table Cut-offs for calculation of intra-protein interactions responsible for protein stability. (PDF)

S3 Table The pairwise comparison matrix derived for calculating priority values for thermostability protein features. (PDF)

S4 Table Dataset of thermostable-mesostable protein pairs test set taken from the RCSB Protein Data Bank. (PDF)

S5 Table Ranking of thermostable mutants of *Bacillus subtilis* lipases by RankProt (Mutants obtained by Acharya et al. 2004 by directed evolution strategies). (PDF)

S6 Table The average number of secondary structures present in each frame after 30ns MD simulation. (PDF)

S1 Fig Scatter plot. Top left: Rank differences obtained through RankProt for the thermostable-mesostable protein pairs test set taken from the RCSB Protein Data Bank. Most of the differences being positive implies that the thermostable protein obtains a higher rank. Top right: Mutant rank differences obtained by RankProt for bacteriophage T4 lysozyme. Most of the differences being positive implies that the thermostable protein obtains a higher rank. Wild type optimum temperature = 40°C; T_m_ = 51.68°C. Bottom: Rank Differences obtained by RankProt for human lysozyme.
(PDF)

S2 Fig Graphical illustration of number of hydrogen bond in wild type and mutated proteins. MM: main chain-main chain; MS: main chain-side chain; SS: Side chain-side chain.
(PDF)

S3 Fig Graphical illustration of the number of hydrogen bonds with distance <3Å in mutants and wild type. (PDF)

S4 Fig Root mean square fluctuation of α-carbon atoms as a function of atoms of 1i6w and its mutants from RMSF study at 320K during the 30ns simulation is shown. A) 1i6w at 320K, B) mut 1 at 320K, C) mut 2 at 320K.
(PDF)

S5 Fig Root mean square fluctuation of α-carbon atoms as a function of atoms of 1i6w and its mutants from RMSF study at 330K during the 30ns simulation is shown. A) 1i6w at 330K, B) mut 1 at 330K, C) mut 2 at 330K.
(PDF)

S6 Fig Root mean square fluctuation of α-carbon atoms as a function of atoms of 1i6w and its mutants from RMSF study at 350K during the 30ns simulation is shown. A) 1i6w at 350K, B) mut 1 at 350K, C) mut 2 at 350K.
(PDF)

S7 Fig *Bacillus subtilis* 168 lipase wild type and mutants in pET28a (5.3 kb) was digested with BamHI and NdeI. The mutants have been designated as mut 1 and mut 2. WTp, 1p and 2p stands for the undigested plasmids of wild type, mut 1 and mut 2 respectively.
(PDF)

S8 Fig SDS PAGE of purified wild type and mutant enzymes. Electrophoresis was carried out on 12.5% polyacrylamide gel. M: Broad range protein molecular weight marker from NEB; WT: Wild type protein. The molecular weight of the wild type and mutant enzymes is 23KD.
(PDF)
