# Supplementary material for: RankProt: A multi criteria-ranking platform to attain protein thermostabilizing mutations and its in vitro applications - Attribute based prediction method on the principles of Analytical Hierarchical Process
Source: PLoS One. 2018 Oct 4;13(10):e0203036. doi: 10.1371/journal.pone.0203036 (PMC6171822; doi:10.1371/journal.pone.0203036)
Supplement: S1 Table — (PDF) [file pone.0203036.s001.pdf]

S1 Table: The 127 thermostable and mesostable protein pairs forming the final dataset

PDB Thermo	Source	Temperature °C	Macromolecule name	PDB Meso	Temperature °C	Macromolecule name
1uz5	*Pyrococcus horikoshii*	98	Molybdopterin biosynthesis moea protein	1bev	38	Bovine enterovirus coat proteins vp1 to vp4
1t1g	*Bacillus sp. Mn-32*	60	Kumamolisin	1bh6	37	Subtilisin dy
1thm	*Thermoactinomyces vulgaris*	50	Thermitase	1bh6	37	Subtilisin dy
2ajr	*Thermotoga maritima*	80	Sugar kinase, pfkb family	1bx4	37	Protein (adenosine kinase)
1nee	*Methanothermobacter thermautotrophicus*	65	Probable translation initiation factor 2 beta subunit	1cf5	20	Protein (beta-momorcharin)
1rfk	*Mastigocladus laminosus*	75	Ferredoxin	1czp	20	Ferredoxin i
1j6r	*Thermotoga maritima*	80	Methionine synthase	1d4m	37	Protein (coxsackievirus a9)
1nz0	*Thermotoga maritima*	80	Ribonuclease P protein component	1d6t	37	Ribonuclease p
1ayg	*Hydrogenobacter thermophilus*	70	Cytochrome C-552	1dvv	30	Cytochrome c551
1clc	*Clostridium thermocellum*	60	Endoglucanase celd; ec: 3.2.1.4	1e1f	20	Beta-glucosidase
1ixk	*Pyrococcus horikoshii*	98	Methyltransferase	1ej0	37	Ftsj

1tzv	*Thermotoga maritima*	80	N utilization substance protein B homolog	1ey1	37	Antitermination factor nusb
1io9	*Sulfolobus solfataricus*	85	Cytochrome p450 cyp119	1f20	37	Nitric-oxide synthase
1lfp	*Aquifex aeolicus*	85	Hypothetical protein aq_1575	1f5n	37	Interferon-induced guanylate-binding protein 1
1mpp	*Rhizomucor pusillus*	50	Pepsin	1fmx	25	Saccharopepsin
1v37	*Thermus thermophilus*	75	Phosphoglycerate mutase	1fzt	24	Phosphoglycerate mutase
1pzx	*Geobacillus stearothermo philus*	55	Hypothetical protein apc36103	1g7n	37	Adipocyte lipid-binding protein
1vkz	*Thermotoga maritima*	80	Phosphoribosylamine--glycine ligase	1gso	37	Protein (glycinamide ribonucleotide synthetase)
1gw0	*Melanocarpu s albomyces*	50	Laccase-1	1gyc	20	Laccase 2
1vp5	*Thermotoga maritima*	80	2,5-diketo-d-gluconic acid reductase	1hw6	30	2,5-diketo-d-gluconic acid reductase
1vku	*Thermotoga maritima*	80	Acyl carrier protein	1hy8	20	Acyl carrier protein
1wr2	*Pyrococcus horikoshii*	98	Hypothetical protein ph1789	1j0n	30	Xanthan lyase
1o5z	*Thermotoga maritima*	80	Folylpolyglutamate synthase/dihydrofolate synthase	1jbw	37	Folylpolyglutamate synthase
1jdq	*Thermotoga maritima*	80	Hypothetical protein tm0983	1je3	37	Hypothetical 8.6 kda protein in amya-flie intergenic region
1zar	*Archaeoglob us fulgidus*	82	Rio2 kinase	1jkk	37	Death-associated protein kinase
1x0t	*Pyrococcus horikoshii*	98	Ribonuclease p protein component 4	1jln	37	Protein tyrosine phosphatase, receptor type, r

1xqo	*Pyrobaculum aerophilum*	100	8-oxoguanine dna glycosylase	1jxo	37	Postsynaptic density protein
1lab	*Geobacillus stearothermo philus*	55	Dihydrolipoamide acetyltransferase	1k8o	37	E2 component of branched-chain alpha-ketoacid dehydrogenase
1v7r	*Pyrococcus horikoshii*	98	Hypothetical protein ph1917	1kfq	28	Phosphoglucomutase 1
1vr8	*Thermotoga maritima*	80	Gtp binding regulator	1kfr	25	Hemoglobin
1jcu	*Methanother mobacter thermautotro phicus*	65	Conserved protein mth1692	1kk9	37	Probable translation factor ycio
1jg1	*Pyrococcus furiosus*	100	Protein-l-isoaspartate o-methyltransferase	1kr5	37	Protein-l-isoaspartate o-methyltransferase
1olr	*Humicola grisea*	45	Endo-beta-1,4-glucanase	1ks4	30	Endoglucanase a
1vcd	*Thermus thermophilus*	75	Ndx1	1ktg	25	Diadenosine tetraphosphate hydrolase
2c4x	*Clostridium thermocellu m*	60	Endoglucanase	1l0q	37	Surface layer protein
1xjk	*Thermotoga maritima*	80	Ribonucleotide reductase, b12-dependent	1l1l	37	Ribonucleoside triphosphate reductase
1ldn	*Geobacillus stearothermo philus*	80	L-lactate dehydrogenase	1ldg	37	L-lactate dehydrogenase
1bqc	*Thermobifida fusca*	45	Protein (beta-mannanase)	1lf1	20	Cel5
1xhc	*Pyrococcus furiosus*	100	Nadh oxidase /nitrite reductase	1m6i	37	Programmed cell death protein 8

1wl7	*Geobacillus thermodenitrificans*	60	Arabinanase-ts	1mdw	37	Calpain ii, catalytic subunit
1oi0	*Archaeoglobus fulgidus*	82	Hypothetical protein af2198	1mqa	37	Integrin alpha-l
2d2e	*Thermus thermophilus*	75	Sufc protein	1mt0	37	Hemolysin secretion atp-binding protein
1kgs	*Thermotoga maritima*	80	Dna binding response regulator d	1mvo	20	Phop response regulator
1i1w	*Thermoascus aurantiacus*	45	Endo-1,4-beta-xylanase	1mzd	37	Pro-granzyme k
1jrm	*Methanothermobacter thermautotrophicus*	65	Conserved hypothetical protein mth637	1n91	37	Orf, hypothetical protein
1iv0	*Thermus thermophilus*	75	Hypothetical protein	1nmn	37	Hypothetical protein yqgf
1urs	*Alicyclobacillus acidocaldarius*	60	Maltose-binding protein	1nnf	37	Iron-utilization periplasmic protein
1l7m	*Methanocaldococcus jannaschii*	85	Phosphoserine phosphatase	1nnl	37	L-3-phosphoserine phosphatase
1wot	*Thermus thermophilus*	75	Putative minimal nucleotidyltransferase	1no5	37	Hypothetical protein hi0073
2etd	*Thermotoga maritima*	80	Lema protein	1nwk	37	Actin, alpha skeletal muscle
1pz3	*Geobacillus stearothermophilus*	55	Alpha-l-arabinofuranosidase	1nxc	37	Mannosyl-oligosaccharide 1,2-alpha-mannosidase ia

1n75	*Thermus thermophilus*	75	Glutamyl-trna synthetase	1nyl	37	Glutaminyl-trna synthetase
1t6c	*Aquifex aeolicus*	85	Exopolyphosphatase	1nyn	25	Hypothetical 12.0 kda protein in nam8-gar1 intergenic region
1iq0	*Thermus thermophilus*	75	Arginyl-trna synthetase	1nzj	37	Hypothetical protein yadb
1t4y	*Synechococcus elongatus*	60	Adaptive-response sensory-kinase sasa	1o2f	37	Pts system, glucose-specific iia component
1zko	*Thermotoga maritima*	80	Glycine cleavage system h protein	1o78	30	Biotin carboxyl carrier protein of methylmalonyl-coa carboxyl-transferase
1z5z	*Sulfolobus solfataricus*	85	Helicase of the snf2/rad54 family	1oyy	37	Atp-dependent dna helicase
1pmh	*Caldicellulosiruptor saccharolyticus*	65	Beta-1,4-mannanase	1oyz	37	Hypothetical protein yiba
1t6t	*Aquifex aeolicus*	85	Putative protein	1pui	37	Probable gtp-binding protein engb
1vbl	*Bacillus sp. Ts-47*	60	Pectate lyase 47	1pxz	20	Major pollen allergen jun a 1
1uet	*Archaeoglobus fulgidus*	82	Trna nucleotidyltransferase	1q6d	20	Beta-amylase
1o98	*Bacillus stearothermophilus*	55	2,3-bisphosphoglycerate-independent phosphoglycerate mutase	1q8k	37	Eukaryotic translation initiation factor 2 subunit 1
1mrz	*Thermotoga maritima*	80	Riboflavin kinase/fmn adenylyltransferase	1q9s	37	Hypothetical protein flj11149
1mgt	*Thermococcus kodakarensis*	95	Protein (o6-methylguanine-dna methyltransferase)	1qnt	37	Methylated-dna--protein-cysteine methyltransferase

2bm3	*Clostridium thermocellum*	60	Scaffolding dockerin binding protein a	1qzn	37	Cellulosomal scaffoldin adaptor protein b
1wf3	*Thermus thermophilus*	75	Gtp-binding protein	1rfl	37	Probable trna modification gtpase trme
1vrx	*Acidothermus cellulolyticus*	60	Endocellulase e1 from a. Cellulolyticus	1rh9	25	Endo-beta-mannanase
1ud9	*Sulfolobus tokodaii*	80	Dna polymerase sliding clamp a	1ri6	37	Putative isomerase ybhe
1ye8	*Aquifex aeolicus*	85	Hypothetical upf0334 kinase-like protein aq_1292	1rkb	37	Protein ad-004
1vjr	*Thermotoga maritima*	80	4-nitrophenylphosphatase	1rkq	37	Hypothetical protein yida
1mxg	*Pyrococcus woesei*	100	Alpha amylase	1rpa	20	Prostatic acid phosphatase
1q0u	*Geobacillus stearothermophilus*	55	Bstdead	1s2m	25	Putative atp-dependent rna helicase dhh1
1lva	*Moorella thermoacetica*	55	Selenocysteine-specific elongation factor	1sjx	38	Immunoglobulin vh domain
1c3p	*Aquifex aeolicus*	85	Protein (hdlp (histone deacetylase-like protein))	1sy1	20	Nitrophorin 4
1y8a	*Archaeoglobus fulgidus*	82	Hypothetical protein af1437	1tf1	37	Negative regulator of allantoin and glyoxylate utilization operons
1tty	*Thermotoga maritima*	80	Rna polymerase sigma factor rpod	1tlh	37	10 kda anti-sigma factor
1t7l	*Thermotoga maritima*	80	5-methyltetrahydropteroyltri	1u22	18	5-methyltetrahydropteroyltriglu

			glutamate--homocysteine methyltransferase			tamate--homocysteine methyltransferase
1pe5	*Bacillus thermoproteolyticus*	60	Thermolysin	1u4g	30	Elastase
1vkc	*Pyrococcus furiosus*	100	Putative acetyl transferase	1u6m	37	Acetyltransferase, gnat family
1zy9	*Thermotoga maritima*	80	Alpha-galactosidase	1uas	20	Alpha-galactosidase
1wj9	*Thermus thermophilus*	75	Crispr-associated protein	1ued	28	P450 monooxygenase
1lf6	*Thermoanaerobacterium thermosaccharolyticum*	60	Glucoamylase	1ulv	30	Glucodextranase
1u4h	*Thermoanaerobacter tengcongensis*	75	Heme-based methyl-accepting chemotaxis protein	1upw	37	Oxysterols receptor lxr-beta
1dq3	*Pyrococcus furiosus*	100	Endonuclease	1v5d	20	Chitosanase
1gh8	*Methanothermobacter thermautotrophicus*	65	Translation elongation factor 1beta	1va9	37	Down syndrome cell adhesion molecule like-protein 1b
2ars	*Thermoplasma acidophilum*	59	Lipoate-protein ligase a	1vqz	37	Lipoate-protein ligase, putative
1qo2	*Thermotoga maritima*	80		1vzw	28	Phosphoribosyl isomerase a
1t95	*Archaeoglobus fulgidus*	82	Hypothetical protein af0491	1w45	37	Annexin a8

1o2d	*Thermotoga maritima*	80	Alcohol dehydrogenase, iron-containing	1wik	37	Thioredoxin-like protein 2
1o7i	*Sulfolobus solfataricus*	85	Single stranded dna binding protein	1wjj	18	Hypothetical protein f20o9.120
1mtz	*Thermoplas ma acidophilum*	59	Proline iminopeptidase	1wm1	30	Proline iminopeptidase
1ujp	*Thermus thermophilus*	75	Tryptophan synthase alpha chain	1wq5	37	Tryptophan synthase alpha chain
1jl2	**Escherichia coli* and *Thermus thermophilus*	75	Chimeric Rnase H	1wsh	37	Ribonuclease hi
1oz9	*Aquifex aeolicus*	85	Hypothetical protein aq_1354	1xax	37	Hypothetical upf0054 protein hi0004
1z0w	*Archaeoglob us fulgidus*	82	Putative protease la homolog type	1xmj	37	Cystic fibrosis transmembrane conductance regulator
1cz4	*Thermoplas ma acidophilum*	59	Vcp-like atpase	1xmv	37	Reca protein
1ryj	*Methanother mococcus thermolithotr ophicus*	65	Unknown	1xs3	37	Hypothetical protein xc975
1in4	*Thermotoga maritima*	80	Holliday junction dna helicase ruvb	1xwi	37	Skd1 protein
1vlm	*Thermotoga maritima*	80	Sam-dependent methyltransferase	1xxl	20	Ycgj protein
1ix5	*Methanother mococcus*	65	Fkbp	1y0o	18	Fkbp-type peptidyl-prolyl cis-trans isomerase 3

	thermolithotrophicus					
2bog	*Thermomonospora fusca*	45	Endoglucanase e-2	1y7m	20	Hypothetical protein bsu14040
1pbt	*Thermotoga maritima*	80	6-phosphogluconolactonase	1y89	28	Devb protein
1vhu	*Archaeoglobus fulgidus*	82	Hypothetical protein af1521	1yd9	37	Core histone macro-h2a.1
1zdr	*Geobacillus stearothermophilus*	55	Dihydrofolate reductase	1yho	37	Dihydrofolate reductase
1ku0	*Geobacillus stearothermophilus*	55	L1 lipase	1ys2	28	Lipase
1sau	*Archaeoglobus fulgidus*	82	Sulfite reductase, desulfoviridin-type subunit gamma	1yx3	25	Hypothetical protein dsrc
1u04	*Pyrococcus furiosus*	100	Hypothetical protein pf0537	1z6t	37	Apoptotic protease activating factor 1
1ilo	*Methanothermobacter thermautotrophicus str. Delta h*	65	Conserved hypothetical protein mth895	1z8f	37	Guanylate kinase
1ytl	*Archaeoglobus fulgidus*	82	Acetyl-coa decarboxylase/synthase complex epsilon subunit 2	1zbe2	38	Coat protein vp1
1wg8	*Thermus thermophilus*	75	Predicted s-adenosylmethionine-dependent methyltransferase	1zq9	37	Probable dimethyladenosine transferase

1z8s	*Geobacillus stearothermophilus*	55	Dna primase	1zrh	37	Heparan sulfate glucosamine 3-o-sulfotransferase 1
1sfs	*Geobacillus stearothermophilus*	55	Hypothetical protein	1zsw	30	Glyoxalase family protein
1ui9	*Thermus thermophilus*	75	Chorismate mutase	2a22	20	Vacuolar protein sorting 29
1kkh	*Methanocaldococcus jannaschii*	85	Mevalonate kinase	2a2d	37	N-acetylgalactosamine kinase
1ihn	*Methanothermobacter thermautotrophicus*	65	Hypothetical protein mth938	2ab1	37	Hypothetical protein
1v3y	*Thermus thermophilus*	75	Peptide deformylase	2ai9	37	Peptide deformylase
1xbi	*Methanocaldococcus jannaschii*	85	50s ribosomal protein l7ae	2aif	25	Ribosomal protein l7a
1ryq	*Pyrococcus furiosus*	100	Dna-directed rna polymerase, subunit e"	2aou	37	Histamine n-methyltransferase
1vhn	*Thermotoga maritima*	80	Putative flavin oxidoreductase	2b0m	37	Dihydroorotate dehydrogenase, mitochondrial
1o0x	*Thermotoga maritima*	80	Methionine aminopeptidase	2b3l	37	Methionine aminopeptidase 1
1nv8	*Thermotoga maritima*	80	Hemk protein	2b3t	37	Protein methyltransferase hemk
1ufk	*Thermus thermophilus*	75	Tt0836 protein	2b3t	37	Protein methyltransferase hemk

1j6o	*Thermotoga maritima*	80	Tatd-related deoxyribonuclease	2b75	37	Lysozyme
1d1n	*Geobacillus stearothermophilus*	55	Initiation factor 2	2crv	37	Translation initiation factor if-2
1v98	*Thermus thermophilus*	75	Thioredoxin	2fch	37	Thioredoxin 1
1brf	*Pyrococcus furiosus*	100	Protein (rubredoxin)	2rdv	30	Rubredoxin
1caa	*Pyrococcus furiosus*	80	Rubredoxin	8rxn	37	Rubredoxin

* This is a thermostable chimera combining the folding core from *T. thermophilus* RNase H and the remaining region of *E. coli* RNase H
