# Supplementary material for: RankProt: A multi criteria-ranking platform to attain protein thermostabilizing mutations and its in vitro applications - Attribute based prediction method on the principles of Analytical Hierarchical Process
Source: PLoS One. 2018 Oct 4;13(10):e0203036. doi: 10.1371/journal.pone.0203036 (PMC6171822; doi:10.1371/journal.pone.0203036)
Supplement: S2 Table — (PDF) [file pone.0203036.s002.pdf]

S2 Table: Cut-off values for calculation of intra-protein interactions responsible for protein stability

**Features**	**Qualifying Criteria**	**Importance in stability**	**Reference**
Hydrophobic interactions	ALA, VAL, LEU, ILE, MET, PHE, TRP, PRO, TYR at a distance of 5 Å	Hydrophobic effect is the dominant driving force in protein folding	Kyte and Dooli ttle, 1982
Disulphide Bridges	Pairs of CYS within 2.2 Å	Covalent bond increases rigidity of protein	Darby et al. 1997
Hydrogen Bonds	Donor-acceptor distance cutoff (oxygen and nitrogen) is 3.50	Increased electrostatic strength	Overington et al 1990
Ionic Interactions	Ionic residue pairs falling with 6Å	Increased electrostatic strength	Vogt et al. 1997
Aromatic-Aromatic Interactions	Pairs of phenyl ring centroids that are epar ated by a preferential distance of between 4.5 to 7 Å account for aromatic interactions	A pair of aromatic interaction contributes between –0.6 and –1.3 kcal/mol to the protein stability (Serrano et al., 1991)	Burley et al 1985
Aromatic-Sulphur Interactions	Interactions between the sulphur atoms of cysteine and methionine and the aromatic rings of phenylalanine, tyrosine and trypto phan within 5.3 Å	Play an important role in protein folding and stabilization.	Reid et al. 1985
Cation-Pi Interactions	When a cationic side chain is near an aromatic side chain within 6 Å separation they account for cation¬π interactions	Play an important role in protein folding and stabilization.	Satyapriya et al. 2004
