# Supplementary material for: RankProt: A multi criteria-ranking platform to attain protein thermostabilizing mutations and its in vitro applications - Attribute based prediction method on the principles of Analytical Hierarchical Process
Source: PLoS One. 2018 Oct 4;13(10):e0203036. doi: 10.1371/journal.pone.0203036 (PMC6171822; doi:10.1371/journal.pone.0203036)
Supplement: S3 Table — (PDF) [file pone.0203036.s003.pdf]

S3 Table: The pairwise comparison matrix derived for calculating priority values for thermostability protein features

**Interactions**	**BT**	**GT**	**IGT**	**HB**	**SB**	**II**	**HI**	**PV**	**NASA**	**PASA**	**C ASA**	**ASI**	**CPI**	**AAI**	**MMH**	**MSH**	**SSH**
BT	1.00	0.40	0.40	0.40	0.40	0.30	0.50	0.80	0.80	3.00	0.30	1.70	0.40	0.80	0.30	0.60	0.60
GT	2.30	1.00	0.90	1.00	0.90	0.78	1.00	1.80	1.80	7.00	0.90	3.50	1.00	1.80	0.80	1.40	1.40
IGT	2.67	1.14	1.00	1.14	1.00	0.89	1.14	2.00	2.00	8.00	1.00	4.00	1.14	2.00	0.90	1.60	1.60
HB	2.33	1.00	0.88	1.00	0.88	0.78	1.00	1.75	1.75	7.00	0.88	3.50	1.00	1.75	0.80	1.40	1.40
SB	2.67	1.14	1.00	1.14	1.00	0.89	1.14	2.00	2.00	8.00	1.00	4.00	1.14	2.00	0.90	1.60	1.60
II	3.00	1.29	1.13	1.29	1.13	1.00	1.29	2.25	2.25	9.00	1.13	4.50	1.29	2.25	1.00	1.80	1.80
HI	2.33	1.00	0.88	1.00	0.88	0.78	1.00	1.75	1.75	7.00	0.88	3.50	1.00	1.75	0.80	1.40	1.40
PV	1.33	0.57	0.50	0.57	0.50	0.44	0.57	1.00	1.00	4.00	0.50	2.00	0.57	1.00	0.44	0.80	0.80
NASA	1.33	0.57	0.50	0.57	0.50	0.44	0.57	1.00	1.00	4.00	0.50	2.00	0.57	1.00	0.44	0.80	0.80
PASA	0.33	0.14	0.13	0.14	0.13	0.11	0.14	0.25	0.25	1.00	0.13	0.50	0.14	0.25	0.11	0.20	0.20
CASA	2.70	1.14	1.00	1.14	1.00	0.89	1.14	2.00	2.00	8.00	1.00	4.00	1.14	2.00	0.90	1.60	1.60
ASI	0.67	0.29	0.25	0.29	0.25	0.22	0.29	0.50	0.50	2.00	0.25	1.00	0.29	0.50	0.22	0.40	0.40
CPI	2.33	1.00	0.90	1.00	0.88	0.78	1.00	1.75	1.75	7.00	0.88	3.50	1.00	1.75	0.80	1.40	1.40
AAI	1.33	0.57	0.50	0.57	0.50	0.44	0.57	1.00	1.00	4.00	0.50	2.00	0.57	1.00	0.44	0.80	0.80
MMH	3.00	1.29	1.13	1.29	1.13	1.00	1.29	2.25	2.25	9.00	1.13	4.50	1.29	2.25	1.00	1.80	1.80
MSH	1.67	0.71	0.63	0.71	0.63	0.56	0.71	1.25	1.25	5.00	0.63	2.50	0.71	1.25	0.60	1.00	1.00
SSH	1.67	0.71	0.63	0.71	0.63	0.56	0.71	1.25	1.25	5.00	0.63	2.50	0.71	1.25	0.60	1.00	1.00

* The pairwise comparison matrix was normalized and priority values or eigen values for each feature was computed
