# Supplementary material for: RankProt: A multi criteria-ranking platform to attain protein thermostabilizing mutations and its in vitro applications - Attribute based prediction method on the principles of Analytical Hierarchical Process
Source: PLoS One. 2018 Oct 4;13(10):e0203036. doi: 10.1371/journal.pone.0203036 (PMC6171822; doi:10.1371/journal.pone.0203036)
Supplement: S4 Table — (PDF) [file pone.0203036.s004.pdf]

S4 Table: Dataset of thermostable-mesostable protein pairs test set taken from the RCSB Protein Data Bank (The data is inclusive of both inside and outside the dataset)

Sl	TP	Temperature (°C)	TP Protein	TP Source	MP
1	1vp9	70	DNA ligase	*Thermus filiformis*	1owo
2	3d2c	65	Lipase	*Bacillus subtilis*	1i6w
3	2vul	75	Gh11 xylanase	*E. Coli*	2vug
4	2ak9	50-90	Subtilisin BPN'	*Bacillus amyloliquefaciens*	1sbt
5	1a5z	50-90	Lactate dehydrogenase	*Thermotoga maritima*	1ldn
6	1b26	75	Glutamate dehydrogenase	*Thermotoga maritima*	1bgv
7	1je0	120	5'-methylthioadenosine phosphorylase	*Sulfolobus solfataricus*	1eu8
8	2zf5	105.4	Glycerol kinase	*Thermococcus kodakarensis kod1*	3pnk
9	1t2n	50	Lipase	*Bacillus subtilis*	1i6w
10	1bxc	75	Xylose isomerase	*Thermus caldophilus*	1bhw
11	1bxz	94	Alcohol dehydrogenase	*Thermoanaerobacter brockii*	1kev
12	1tmy	80	Chey	*Thermotoga maritima*	3chy
13	1bxb	90	Xylose isomerase	*Thermus thermophilus*	1qti
14	1xyz	60	Xylanhydrolase	*Clostridium thermocellum*	2exo
15	1thl	80	Neutral protease	*Bacillus thermoproteolyticus*	1npc
16	2prd	73	Hydrolase	*Thermus thermophilus*	1ino
17	1gtm	75-100	Glutamate dehydrogenase	*Pyrococcus furiosus*	1hrd
18	3pfk	53	Phosphofructose kinase	*Geobacillus stearothermophilus*	2pfk
19	3mds	60	Maganese superoxide dismutase	*Thermus thermophilus*	1qnm
20	1lnf	86	Thermolysin	*Bacillus thermoproteolyticus*	1npc
21	1bdm	90	Malate dehydrogenase	*Thermus thermophilus*	4mdh
22	1a2z	83	Pyrrolidone carboxyl peptidase	*Thermococcus litoralis*	1-aug
23	1a53	70-80	Indole-3-glycerolphosphate synthase	*Sulfolobus solfataricus*	1pii
24	1a5z	80	Lactate dehydrogenase	*Thermotoga maritima*	9ldt
25	1a8h	75	Methionyl-trna synthetase	*Thermus thermophilus*	1qtt
26	1b69	83	Histone hmfa	*Methanothermus fervidus*	1aoi
27	1bjw	75	Aspartate aminotransferase	*Thermus thermophilus*	1bw0
28	1bmd	70	Malate dehydrogenase	*Thermus aquaticus flavus*	1b8p
29	1bvu	83	Glutamate dehydrogenase	*Thermococcus litoralis*	1hrd
30	1bxb	75	Xylose isomerase	*Thermus thermophilus*	1xif
31	1c3u	80	Adenylosuccinate lyase	*Thermotoga maritima*	1auw
32	1coj	85	Superoxide dismutase	*Aquifex pyrophilus*	1var
33	1dv7	65	Orotidine 5V-phosphate decarboxylase	*Methanothermobacter thermoautot*	1eix
34	1ffh	70	Gtpase domains of the signal sequence	*Thermus aquaticus*	1fts
35	1gln	75	Glutamyl-trna synthetase	*Thermus thermophilus*	1euq
36	1gtm	97-100	Glutamate dehydrogenase	*Pyrococcus furiosus*	1aup

37	1sss	70-85	Iron superoxide dismutase	*Sulfolobus solfataricus*	1qnn
38	1xgs	97-100	Methionine aminopeptidase	*Pyrococcus furiosus*	1mat
39	1ykf	65	NADP-dependent alcohol dehydrogenase	*Thermoanaerobium brockii*	1kev
40	1yna	48	Endo-1,4-beta-xylanase	*Thermomyces lanuginosus*	1xnd
41	2btm	60	Triosephosphate isomerase	*Geobacillus stearothermophilus*	1tpf
42	4pfk	55	Phosphofructokinase	*Geobacillus stearothermophilus*	1pfk
43	1iqz	52	Ferredoxin	*Bacillus thermoproteolyticus*	1fca
44	1hjs	50	Fungal beta-1,4-galactanases	*Thielavia heterothallica*	1zqd
45	1uek	75	4-(cytidine 5'-diphospho)-2c-methyl-d- erythritol kinase	*Thermus thermophilus*	1dqj
46	1wfr	75	Conserved hypothetical protein tt1886, possibly sterol carrier protein, from thermus thermophilus hb8	*Thermus thermophilus hb8*	1x4v
47	1yz7	103	C-terminal segment of alpha subunit of aif2 from pyrococcus abyssi	*Pyrococcus abyssi*	1a1a
48	1vrm	80	Hypothetical protein (tm1553) from thermotoga maritima	*Thermotoga maritima msb8*	1n7j
49	1a76	85	5'-3' exo/endo nuclease	*Methanococcus jannaschii*	1ut8
50	1v6s	75	Phosphoglycerate kinase	*Thermus thermophilus hb8*	1puz
51	1jji	82	Carboxylesterase	*Archaeon archaeoglobus*	1k4y
52	1pjr	55	Dna helicase	*Bacillus stearothermophilus*	1zrr
53	1lf6	60	Glucoamylase	*Thermoanaerobacterium thermosaccharolyticum*	1ulv
54	1js4	45	Endo/exocellulase:cellobiose	*Thermomonospora fusca*	1kfq
55	1woy	75	Methionyl trna synthetase y225f mutant	*Thermus thermophilus*	1l1l
56	1vmb	80	30s ribosomal protein s6 (tm0603)	*Thermotoga maritima*	1wjh
57	1yqe	82	Conserved hypothetical protein af0625	*Archaeoglobus fulgidus*	1hdh
58	1esw	70	Amylomaltase	*Thermus aquaticus*	1yht
59	1im5	98	Alpha-glucosidase (tm0752)	*Thermotoga maritima*	1frr
60	1vjt	80	Alpha-glucosidase (tm0752)	*Thermotoga maritima*	2bw0
61	1bqc	45	Beta-mannanase	*Thermomonospora fusca*	1lf1
62	2bog	45	Catalytic domain of endo-1,4-glucanase cel6a mutant y73s	*Thermobifida fusca*	1y7m
63	1i1w	45	Thermostable xylanase	*Thermoascus aurantiacus*	1mzd
64	1olr	45	Humicola grisea cel12a enzyme structure	*Humicola grisea*	1ks4
65	1d1n	55	Fmet-trnafmet binding domain of becillus stearothermophillus translation initiation factor if2	*Bacillus stearothermophillus*	2crv
66	2ars	59	Lipoate-protein ligase a	*Thermoplasma acidophilum*	1vqz
67	1wl7	60	Thermostable arabinanase	*Bacillus thermodenitrificans*	1mdw
68	1vbl	60	Thermostable pectate lyase pl 47	*Bacillus sp. Ts-47*	1pxz

69	1t1g	60	Mutant e23a of kumamolisin, a sedolisin type proteinase (previously called kumamolysin or kscp)	*Bacillus sp. Mn-32*	1bh6
70	1clc	60	Endoglucanase D	*Clostridium Thermocellum 1clc*	1eif
71	1v3y	75	Peptide deformylase from thermus thermophilus hb8	*Thermus thermophilus*	2ai9
72	1ujp	75	Tryptophan synthase a-subunit from thermus thermophilus hb8	*Thermus thermophilus*	1wq5
73	1n75	75	Glutamyl-trna synthetase	*Thermus thermophilus*	1nyl
74	1v37	75	Phosphoglycerate mutase	*Thermus thermophilus hb8*	1fzt
75	1v35	75	Crystal Structure of Eoyl-ACP Reductase with NADH	*Plasmodium falciparum*	2ai9
76	1vjr	80	4-nitrophenylphosphatase (tm1742)	*Thermotoga maritima*	1rkq
77	1o2d	80	Alcohol dehydrogenase, iron-containing (tm0920)	*Thermotoga maritima*	1wik
78	1vkz	80	Phosphoribosylamine--glycine ligase (tm1250)	*Thermotoga maritima*	1gso
79	1o5z	80	Folylpolyglutamate synthase (tm0166)	*Thermotoga maritima*	1jbw
80	1qo2	80	Isomerase	*Thermotoga maritima*	1vzw
81	1jdq	80	Tm006 protein	*Thermotoga maritima*	1je3
82	1vku	80	Acyl carrier protein (TM0175)	*Thermotoga maritima*	1hy8
83	1z0w	82	Lon proteolytic domain	*Archaeoglobus fulgidus*	1xmj
84	1io9	85	Oxidoreductase	*Sulfolobus solfataricus*	1f20
85	1c3p	85	Hdac homolog	*Aquifex aeolicus*	1sy1
86	1t6t	85	Putative protein	*Aquifex aeolicus*	1puib
87	1t6c	85	Putative protein	*Aquifex aeolicus*	1nyn
88	1z5z	85	Swi2/snf2 atpase c-terminal domain	*Sulfolobus solfataricus p2*	1oyy
89	1l7m	85	Phosphoserine phosphatase (pi complex)	*Methanococcus jannaschii*	1nnl
90	1wr2	98	Ph1788	*Pyrococcus horikoshii ot3*	1j0n
91	1v7r	98	Nucleotide triphosphate pyrophosphatase	*Pyrococcus horikoshii ot3*	1kfq
92	1mxg	100	(Ca,zn)-dependent alpha-amylase	*Pyrococcus woesei*	1rpa
93	1u04	100	Full length argonaute	*Pyrococcus furiosus dsm 3638*	1z6t
94	1dq3	100	An archaeal intein-encoded homing endonuclease pi-pfui	*Pyrococcus furiosus*	1v5d
95	1xqo	100	Pa-agog, 8-oxoguanine dna glycosylase	*Pyrobaculum aerophilum*	1jxo
96	1brf	100	Rubredoxin (wild type)	*Pyrococcus furiosus*	2rdv
97	1sfs	55	Uncharacterized	*Bacillus Stearothermophilus*	1zsw
98	1lab	55	Lipoyl domain pyruvate dehydrogenase multienzyme complex.	*Bacillus stearothermophilus*	1k8o
99	1mgt	95	O6-methylguanine-dna methyltransferase	*Pyrococcus kodakaraensis*	1qnt

100	1ixk	98	Methyltransferase homolog protein	*Pyrococcus horikoshii*	1ej0
