# Supplementary material for: RankProt: A multi criteria-ranking platform to attain protein thermostabilizing mutations and its in vitro applications - Attribute based prediction method on the principles of Analytical Hierarchical Process
Source: PLoS One. 2018 Oct 4;13(10):e0203036. doi: 10.1371/journal.pone.0203036 (PMC6171822; doi:10.1371/journal.pone.0203036)
Supplement: S5 Table — (PDF) [file pone.0203036.s005.pdf]

S5 Table: Ranking of thermostable mutants of *Bacillus subtilis* lipases by RankProt (Mutants obtained by Acharya et al. 2004 by directed evolution strategies)

Sl. No	Mutant	Rank difference	Temperature °C	No of mutations	Mutations	Method of mutation
1	1T2N	0.093	55	3	L114P, A132D, N166Y	Error-prone PCR
2	1T4M	0.083	55	2	A132D, N166Y	Error-prone PCR
3	3D2C	0.147	60	9	A15S, F17S, A20E, N89Y, G111D, L114P, A132D, I157M, N166Y	Directed evolution
4	3QMM	0.133	78	12	A15S,F17S,A20E,N89Y, G111D,L114P,A132D,M134E,M137P,I157M,S163P,N166Y	Directed evolution
5	3QZU	0.075	60	7	R33Q, D34N, K35D, K112D, M134D, Y139C, I157M	Iterative saturation mutagenesis with randomization sites chosen on the basis of the highest B-factors

*The method is a relative ranking strategy with the sum of the ranks equal to 1, therefore the mesostable counterpart 1I6W has different ranks in each case when compared to its thermostable counterparts.

Reference

Acharya P, Rajakumara E, Sankaranarayanan R, Rao NM. Structural basis of selection and thermostability of laboratory evolved Bacillus subtilis lipase. J Mol Biol. 2004; 3415: 1271-81.
