# Supplementary material for: RankProt: A multi criteria-ranking platform to attain protein thermostabilizing mutations and its in vitro applications - Attribute based prediction method on the principles of Analytical Hierarchical Process
Source: PLoS One. 2018 Oct 4;13(10):e0203036. doi: 10.1371/journal.pone.0203036 (PMC6171822; doi:10.1371/journal.pone.0203036)
Supplement: S6 Table — (PDF) [file pone.0203036.s006.pdf]

S6 Table: The average number of secondary structures present in each frame after 30ns MD simulation

Temperature	Structure	Coil	β--Sheet	β-Bridge	Bend	Turn	α-Helix	3^{10}-Helix
320k	1i6w	0.21	0.19	0.02	0.14	0.11	0.26	0.06
	mut 1	0.22	0.19	0.02	0.14	0.21	0.27	0.05
	mut 2	0.24	0.19	0.02	0.13	0.15	0.27	0.04
330k	1i6w	0.25	0.18	0.02	0.13	0.12	0.26	0.06
	mut 1	0.24	0.17	0.02	0.14	0.13	0.25	0.05
	mut 2	0.25	0.17	0.02	0.15	0.13	0.25	0.05
350k	1i6w	0.23	0.18	0.03	0.14	0.16	0.2	0.06
	mut 1	0.22	0.17	0.02	0.15	0.15	0.24	0.05
	mut 2	0.22	0.18	0.02	0.14	0.13	0.26	0.05
