# Supplementary material for: RankProt: A multi criteria-ranking platform to attain protein thermostabilizing mutations and its in vitro applications - Attribute based prediction method on the principles of Analytical Hierarchical Process
Source: PLoS One. 2018 Oct 4;13(10):e0203036. doi: 10.1371/journal.pone.0203036 (PMC6171822; doi:10.1371/journal.pone.0203036)
Supplement: S1 Fig — Top left: Rank differences obtained through RankProt for the thermostable-mesostable protein pairs test set taken from the RCSB Protein Data Bank. Most of the differences being positive implies that the thermostable protein obtains a higher rank. Top right: Mutant rank differences obtained by RankProt for bacteriophage T4 lysozyme. Most of the differences being positive implies that the thermostable protein obtains a higher rank. Wild type optimum temperature = 40°C; Tm = 51.68°C. Bottom: Rank Differences obtained by RankProt for human lysozyme. (PDF) [file pone.0203036.s007.pdf]

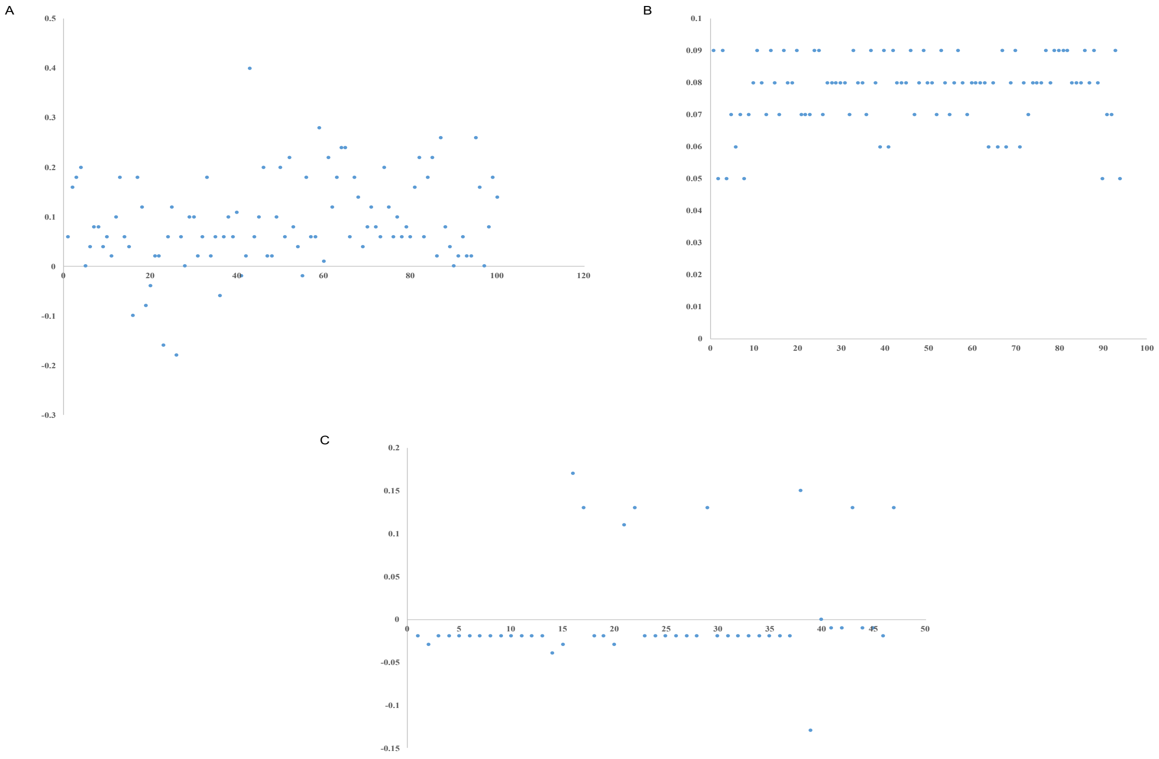

S1 Fig. Scatter plot. Top left: Rank differences obtained through RankProt for the thermostable-mesostable protein pairs test set taken from the RCSB Protein Data Bank. Most of the differences being positive implies that the thermostable protein obtains a higher rank. Top right: Mutant rank differences obtained by RankProt for bacteriophage T4 lysozyme. Most of the differences being positive implies that the thermostable protein obtains a higher rank. Wild type optimum temperature = 40°C; T_m = 51.68^{o}C. Bottom: Rank Differences obtained by RankProt for human lysozyme.
