# Supplementary material for: RankProt: A multi criteria-ranking platform to attain protein thermostabilizing mutations and its in vitro applications - Attribute based prediction method on the principles of Analytical Hierarchical Process
Source: PLoS One. 2018 Oct 4;13(10):e0203036. doi: 10.1371/journal.pone.0203036 (PMC6171822; doi:10.1371/journal.pone.0203036)
Supplement: S2 Fig — MM: main chain-main chain; MS: main chain-side chain; SS: Side chain-side chain. (PDF) [file pone.0203036.s008.pdf]

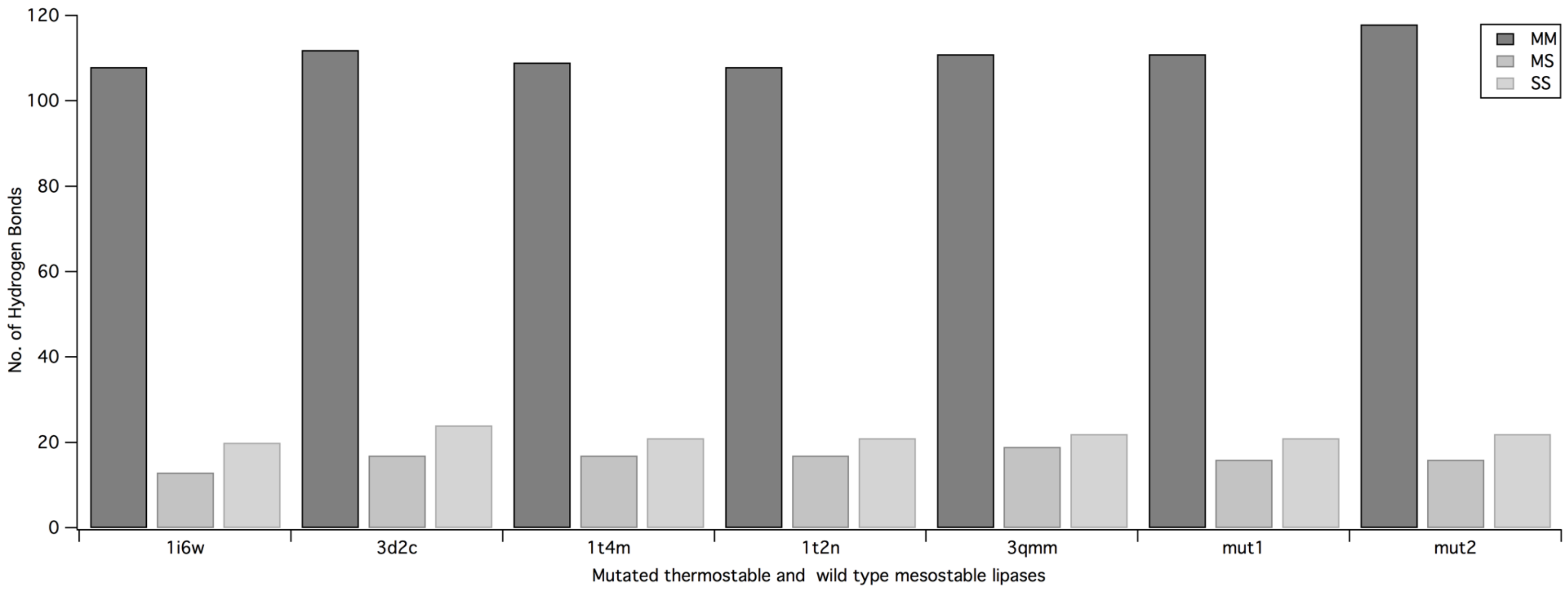

S2 Fig. Graphical illustration of number of hydrogen bond in wild type and mutated proteins. MM: main chain-main chain; MS: main chain-side chain; SS: Side chain-side chain.
