# Supplementary material for: RankProt: A multi criteria-ranking platform to attain protein thermostabilizing mutations and its in vitro applications - Attribute based prediction method on the principles of Analytical Hierarchical Process
Source: PLoS One. 2018 Oct 4;13(10):e0203036. doi: 10.1371/journal.pone.0203036 (PMC6171822; doi:10.1371/journal.pone.0203036)
Supplement: S3 Fig — (PDF) [file pone.0203036.s009.pdf]

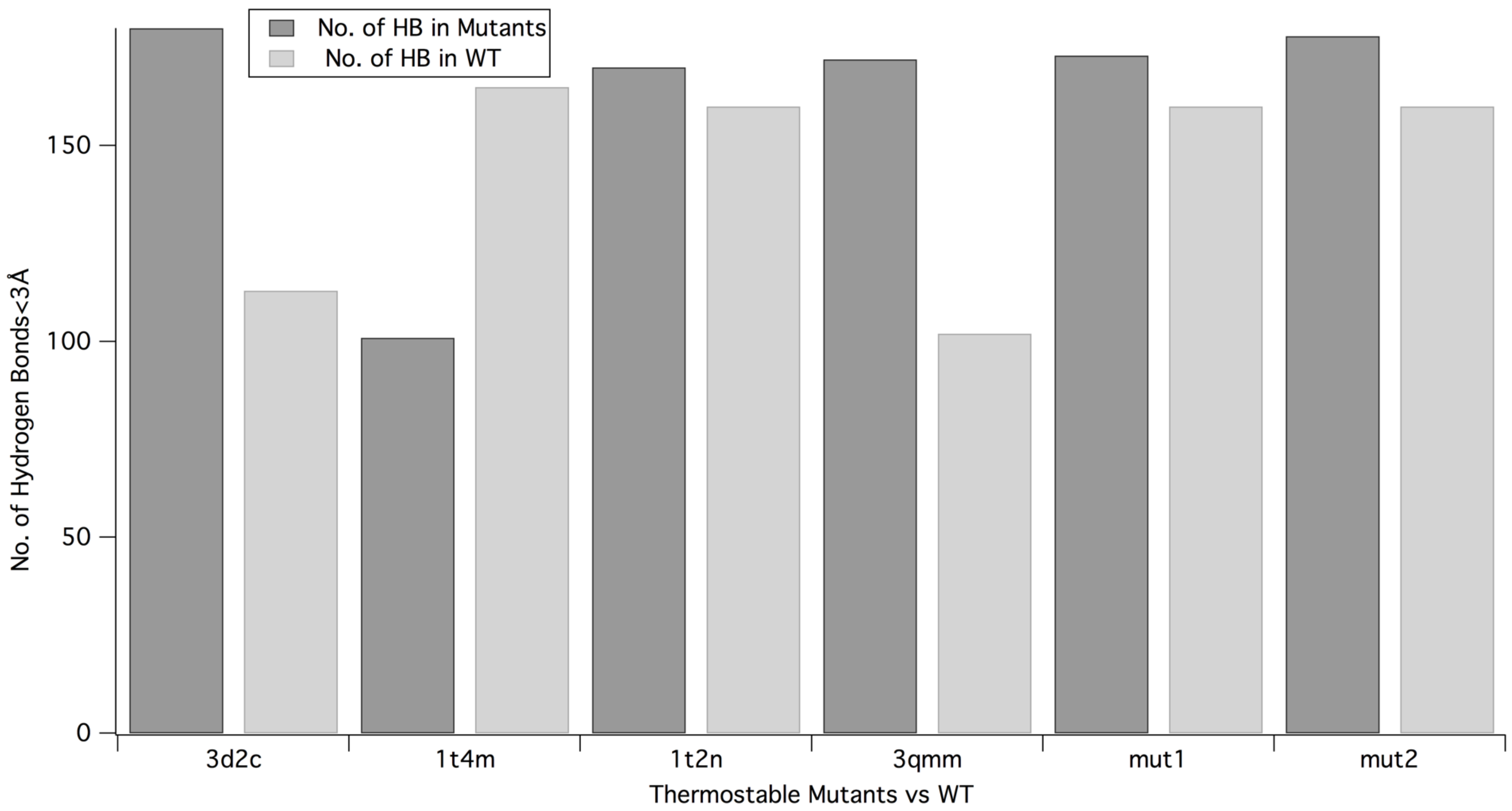

S3 Fig. Graphical illustration of the number of hydrogen bonds with distance <3Å in mutants and wild type.
