# Supplementary material for: RankProt: A multi criteria-ranking platform to attain protein thermostabilizing mutations and its in vitro applications - Attribute based prediction method on the principles of Analytical Hierarchical Process
Source: PLoS One. 2018 Oct 4;13(10):e0203036. doi: 10.1371/journal.pone.0203036 (PMC6171822; doi:10.1371/journal.pone.0203036)
Supplement: S4 Fig — A) 1i6w at 320K, B) mut 1 at 320K, C) mut 2 at 320K. (PDF) [file pone.0203036.s010.pdf]

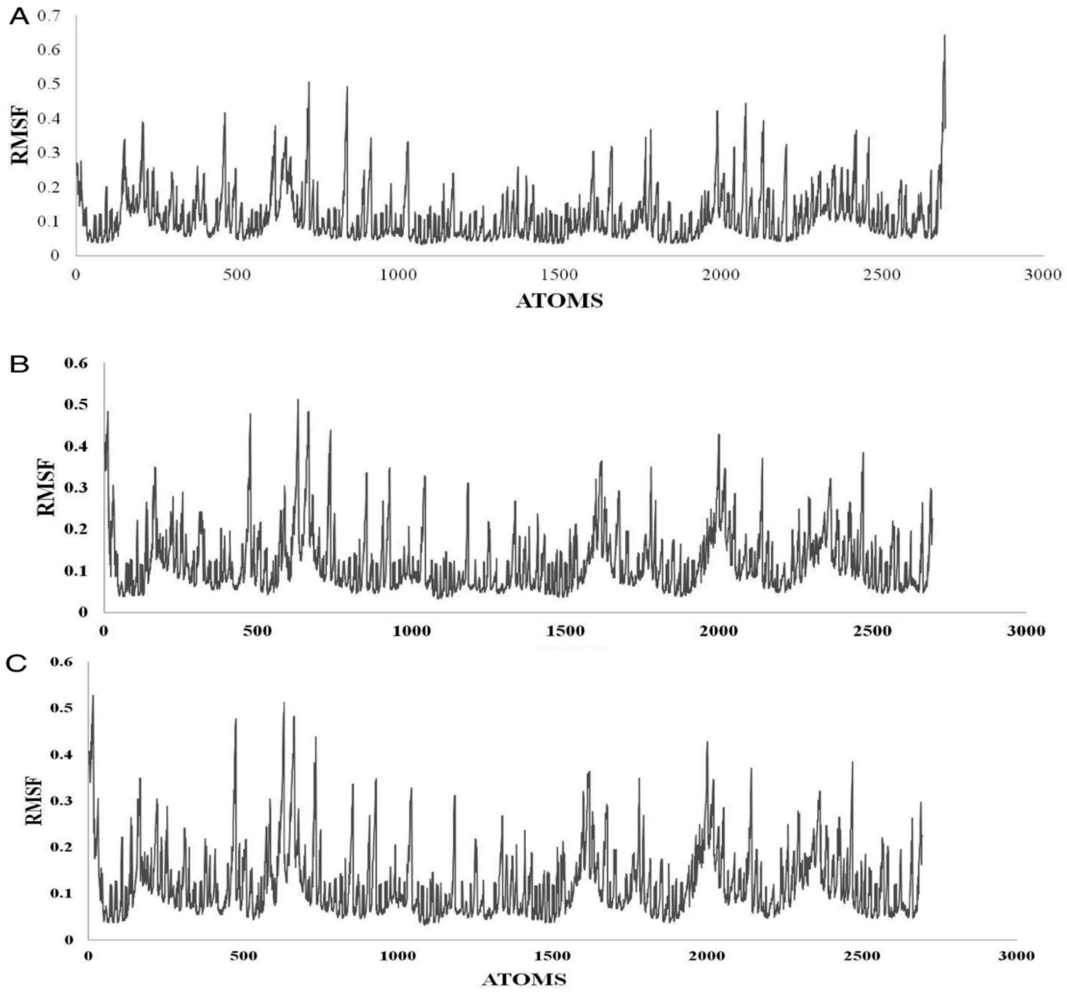

S4 Fig. Root mean square fluctuation of α-carbon atoms as a function of atoms of 1i6w and its mutants from RMSF study at 320K during the 30ns simulation is shown. A) 1i6w at 320K, B) mut 1 at 320K, C) mut 2 at 320K.
