# Supplementary material for: RankProt: A multi criteria-ranking platform to attain protein thermostabilizing mutations and its in vitro applications - Attribute based prediction method on the principles of Analytical Hierarchical Process
Source: PLoS One. 2018 Oct 4;13(10):e0203036. doi: 10.1371/journal.pone.0203036 (PMC6171822; doi:10.1371/journal.pone.0203036)
Supplement: S7 Fig — The mutants have been designated as mut 1 and mut 2. WTp, 1p and 2p stands for the undigested plasmids of wild type, mut 1 and mut 2 respectively. (PDF) [file pone.0203036.s013.pdf]

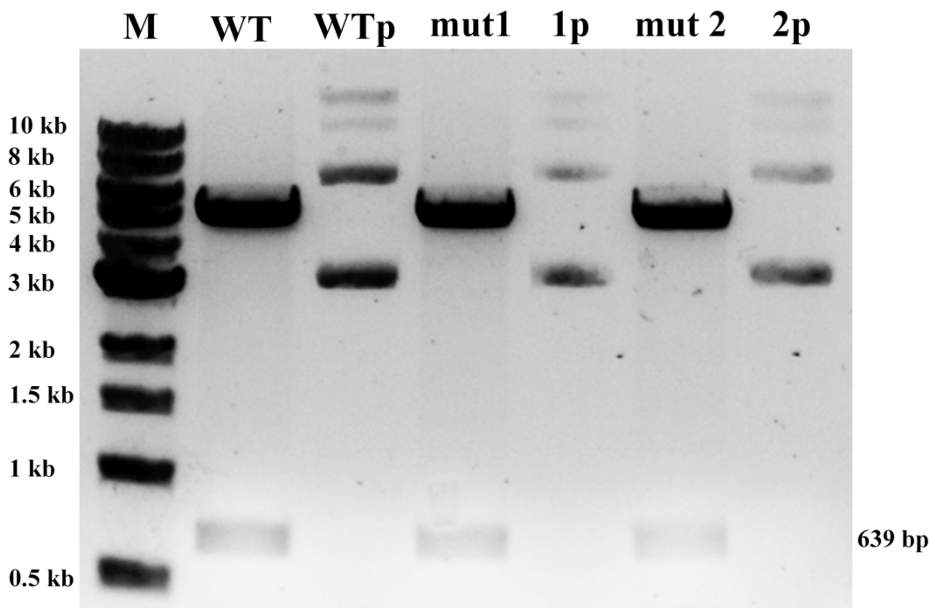


**S7 Fig.** *Bacillus subtilis* 168 lipase wild type and mutants in pET28a (5.3 kb) was digested with BamHI and NdeI. The mutants have been designated as mut 1 and mut 2. WTp, 1p and 2p stands for the undigested plasmids of wild type, mut 1 and mut 2 respectively.
