# Supplementary material for: RankProt: A multi criteria-ranking platform to attain protein thermostabilizing mutations and its in vitro applications - Attribute based prediction method on the principles of Analytical Hierarchical Process
Source: PLoS One. 2018 Oct 4;13(10):e0203036. doi: 10.1371/journal.pone.0203036 (PMC6171822; doi:10.1371/journal.pone.0203036)
Supplement: S8 Fig — Electrophoresis was carried out on 12.5% polyacrylamide gel. M: Broad range protein molecular weight marker from NEB; WT: Wild type protein. The molecular weight of the wild type and mutant enzymes is 23KD. (PDF) [file pone.0203036.s014.pdf]

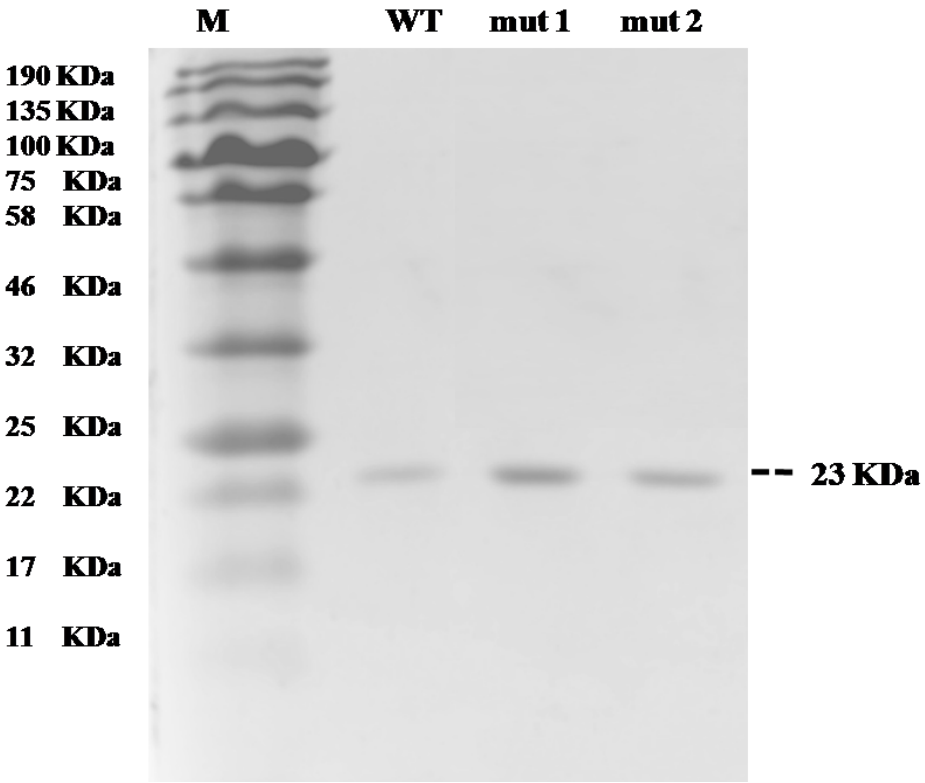

S8 Fig. SDS PAGE of purified wild type and mutant enzymes. Electrophoresis was carried out on 12.5% polyacrylamide gel. M: Broad range protein molecular weight marker from NEB; WT: Wild type protein. The molecular weight of the wild type and mutant enzymes is 23KDa.
